# Record Carrier Diffusion Lengths in Large, Dense Nonfullerene Electron Acceptor Crystals Grown from Polar Aromatic Solvents

**DOI:** 10.1021/jacs.6c06671

**Published:** 2026-07-06

**Authors:** Tamir Halevi, Thomas A. L. Romain, Paul A. Hume, Peter N. Horton, Simon J. Coles, Robert L. Harniman, Ava Blandford, James A. Smith, Richard Cousins, Dominic Alibhai, Andrew J. Orr-Ewing, Simon R. Hall, Michael B. Price

**Affiliations:** † School of Chemistry, 1980 University of Bristol, Bristol BS8 1TS, United Kingdom; ‡ School of Chemical and Physical Sciences, 8491 University of Wellington, Wellington 6012, New Zealand; § National Crystallography Service, 7423 University of Southampton, Southampton SO17 1BJ, United Kingdom; ∥ Nanoscale and Microscale Research Centre, 6123 University of Nottingham, Nottingham NG7 2RD, United Kingdom; ⊥ Wolfson Bioimaging Facility, University of Bristol, Bristol BS8 1TD United Kingdom

## Abstract

Semicrystalline films of nonfullerene acceptors (NFAs) attract considerable interest for optoelectronic applications. Organic single crystals promise superior optoelectronic properties to films; however, NFA crystals have been little used in applications because of their challenging growth and micrometer-nanometer sizes. Here, phenol and acetophenone are used as aromatic, halogen-free recrystallization solvents, resulting in the growth of millimeter-sized Y6 crystals of varying polymorphisms—a crystallization technique which generalizes to other NFA molecules. The crystals show a novel structure which is denser than any previously reported for Y6. We use power-dependent fluorescence and fluorescence microscopy to demonstrate enhanced exciton diffusion lengths (70 ± 15 nm) and novel photophysical responses in these crystals compared to thin films, and compare these properties to quantum chemical calculations. This work demonstrates how material processing can improve future optoelectronics and emphasizes the critical role of molecular packing in current high-performance NFA devices.

## Graphical abstract

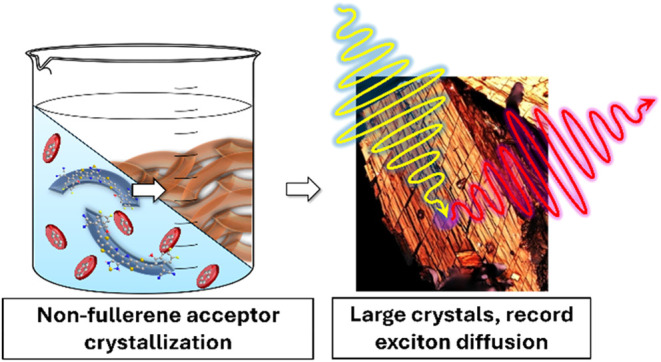

## 1 Introduction

Organic semiconductors are attractive for optoelectronic devices due to their potential for low temperature processability, low cost, and flexibility.^1,2^ Nonfullerene acceptors (NFAs) have been responsible for pushing organic photovoltaic (OPV) device efficiencies to new records. In particular, the Y-series acceptors—structural derivatives of Y6 (also known as BTP-4F)—are among the leading candidates for OPVs,^3^ achieving power conversion efficiencies (PCE) of over 20%,^4^ facilitated by their high charge carrier mobility and favorable blend morphologies. Y6 has been successfully used in photocatalytic hydrogen evolution.^5,6^ Its red absorption spectrum makes it suitable for semitransparent OPVs (i.e., “solar windows”)^7^ and for near-infrared LEDs,^8^ and it has shown promisingly high charge mobilities in organic field effect transistors (OFETs).^7^

Historically, well-studied organic semiconductors such as rubrene and pentacene have showed superior OFET performance with single crystals compared to amorphous materials; in the crystals, the greater structural and energetic order, and lower defect densities improve the charge transport.^9,10^ Only two reports exist so far of single crystal OFETs from Y-series acceptors, with both reporting excellent mobility values.^11,12^ This scarcity is likely due to the difficulty in growing crystals of the Y-series acceptors, which have long flexible alkyl chains.^13^ To date, seven different single crystal structures of Y6 have been reported (most with suboptimal data quality due to the challenging nature of the crystal samples), which have all been grown from some combination of a halogenated solvent (chloroform, chlorobenzene or dibromomethane) and an antisolvent (acetone, methanol, ethanol, isopropanol, or hexane).^12,14–18^

Solvents with different properties are known to encourage the formation of different crystal structures, but the previously published methods have explored only a limited solvent space.^13^ Modeling predicts that different single-crystal structures of Y6 will show different properties, because of the differences in their packing (e.g., different π–π stacking interactions, packing distances and orientations).^19^ Phenol as a solvent component has been demonstrated to produce polymorph selectivity in organic crystals, including the formation of polymorphs that are otherwise hard to access.^20,21^ Although it is a solid at room temperature, it readily forms deep eutectic solutions with certain materials, and in the absence of these unique interactions it may be used as a solvent under mild heating thanks to its moderate volatility and low melting point. As it is both a hydrogen-bond donor (HBD) and halogen-free, its use as a recrystallization solvent for Y6 allows the investigation of a new solvent space. Additionally, its slightly polar aromatic character encourages favorable interactions with Y6 primarily via π–π stacking interactions—demonstrated by the fact that hexane (analogous to the long alkyl chains on Y6) is freely soluble in chloroform, yet is almost insoluble in phenol. In turn, this means use of phenol may favor the formation of Y6 crystal structures with more ordered packing.

Aromatic additives have been shown to improve the performance of Y6-based OPV devices by increasing the crystallinity and improving the morphology of bulk heterojunction films. They are known to associate primarily with the end groups on Y6.^22^ Some of the highest performing devices have utilized additives with hydroxy or ketone groups. For example, Han et al.^23^ used dibenzoylmethane (see Figure 1 (inset)) to prepare Y6:PM6 devices with record PCEs of over 19%. The additive also improved the performance of leading Y6 derivatives including L8-BO and N3, suggesting its generality. Similarly, Yang et al.^24^ used oxybenzone as an additive and achieved a PCE of 17.0%, compared to 15.9% with 1-chloronaphthalene. However, these additives have not yet been explored as solvents in their own right. Although thermal annealing has been demonstrated to be effective at removal of these additives, higher volatility is required when using the materials as a solvent in order to remove them in reasonable time scales.

**Figure 1:**
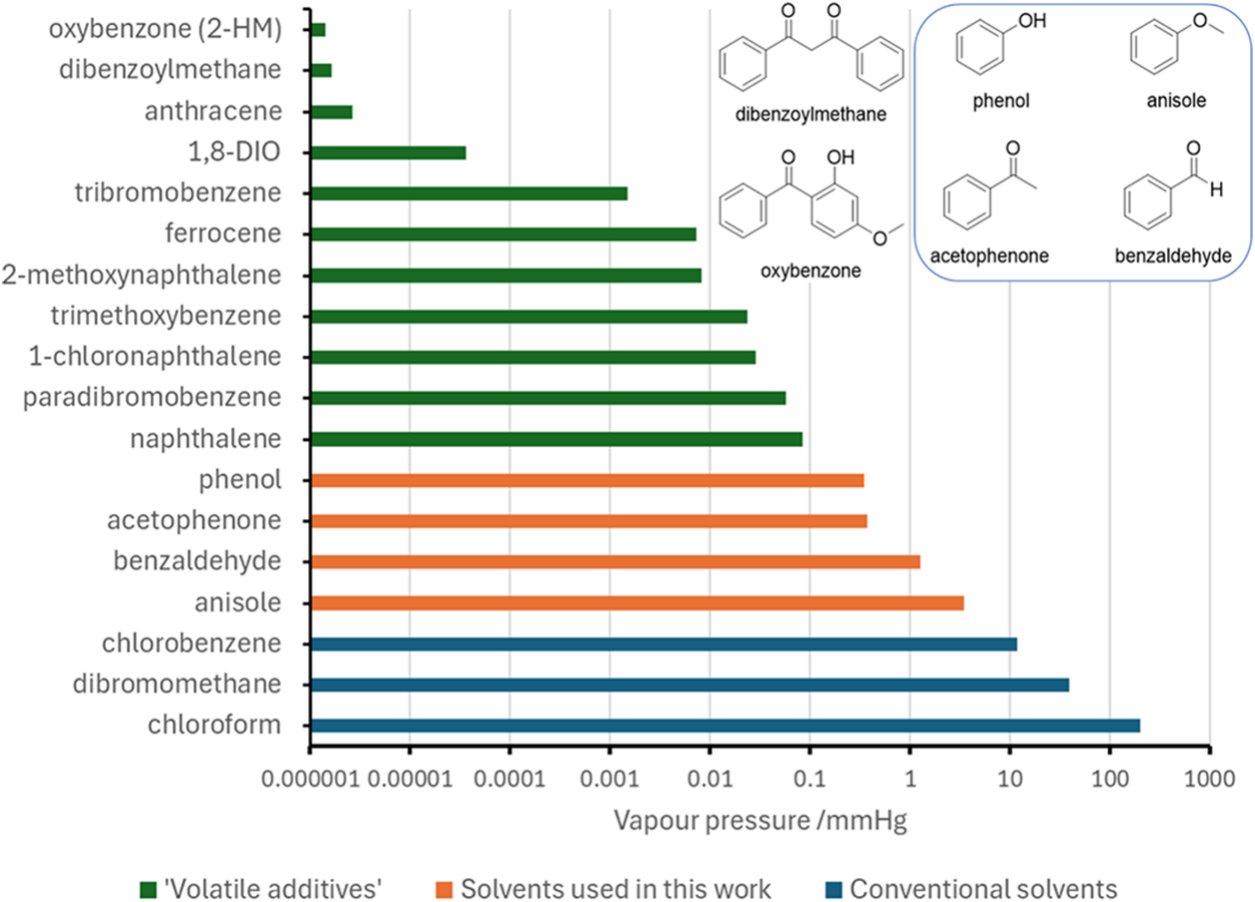
Volatility of chemicals used in the processing of Y6. Inset—structures of additives used in the literature, and, in the blue box, those used as solvents in this work. Vapor pressures obtained from the literature (See Supporting Information Section 10, Table 6 for references), at 25 °C.

Semicrystalline films will retain similarities to single crystal structures in their crystal packing motifs.^25^ Thus, despite the difficulty in crystal growth, single crystal structures remain important in order to offer insight into fundamental properties of device-relevant films. These insights are of particular significance where there are still uncertainties in the literature, for example over the extent to which photoexcitations in neat Y6 films can intrinsically dissociate.^26–28^

Here, we select small aromatic molecules that are volatile enough to be conveniently used as solvents, but “nonvolatile” enough (see Figure 1) to afford the growth of large, high-quality crystals of Y6 and other nonfullerene acceptors by evaporation. Through this new and relatively fast crystallization technique, we show the growth of multiple new Y6 crystalline polymorphs. The larger of these, we study with detailed X-ray crystallography—showing near single-crystalline structural properties, and a novel crystal structure with density higher than previous reports. The size of these crystals allows the first spectroscopic investigations of their optoelectronic properties. We characterize the crystals’ facet and polymorph-dependent fluorescence properties through photoluminescence microscopy. Atomic force microscopy (AFM) shows smooth surfaces and conducting AFM shows potential preferential charge transport channels. Finally, a combination of fluorescence lifetime microscopy, and pulsed intensity-dependent photoluminescence quantum yield (PLQY) measurements shows superior exciton diffusion lengths in some crystals (up to 70 nm), even after exposure to ambient conditions. The pulsed PLQY measurements provide evidence for efficient singlet-state generation via triplet–triplet annihilation compared to thin films. These results highlight the importance of crystal structure/stacking for studying bilayer and bulk-heterojunction OPVs, as well as showcasing the potential gains from harnessing single crystals for devices.

## 2 Results and Discussion

### 2.1 Dissolution and Crystallization

Y6 was dissolved in the solvents phenol, acetophenone, anisole and benzaldehyde, at concentrations from 0.1 to 5 mg/mL. The resulting solutions were then evaporated in watch glasses in an oven at 50–70 °C. The crystals formed in concentric bands reminiscent of Liesegang rings (see Supporting Information Figure S1), which occur due to periodic precipitation/crystallization.^29,30^ In this case, based on the crystallization patterns, we deduce that the periodic bands form by a postnucleation mechanism, where Ostwald ripening leads to the growth of larger crystals.^31^ Crystals were also grown by mild heating (convection) of Y6 in solution and allowing the solution to cool (see Experimental Section, and Supporting Information Figure S4 for details), offering another facile technique for generating large crystals. Over the course of the crystallization experiments, multiple polymorphs were observed (Figure 2a–d): metallic red types of discrete or ensemble “cubic”-like crystals (∼150 by 250 μm) or discrete rod like crystals (∼250 by 20 μm); purple parallelogram-shaped crystals (∼200 by 50 μm); and large “spherulitic”-type platelets (∼2000 by 500 μm). The “metallic” appearance of some of these crystals suggests high optical quality and conductivity. Of the red, “cubic” type metallic/shiny crystals—which we call “metallic red crystals” (MRCs), the largest discrete crystals were selected for optical microscopy and fluorescence microscopy (with ensembles of these used for pulsed photoluminescence quantum yield measurements), and the largest discrete crystals of these were analyzed by X-ray crystallography. For further descriptions of the other observed polymorphs see Supporting Information Section 2.

**Figure 2:**
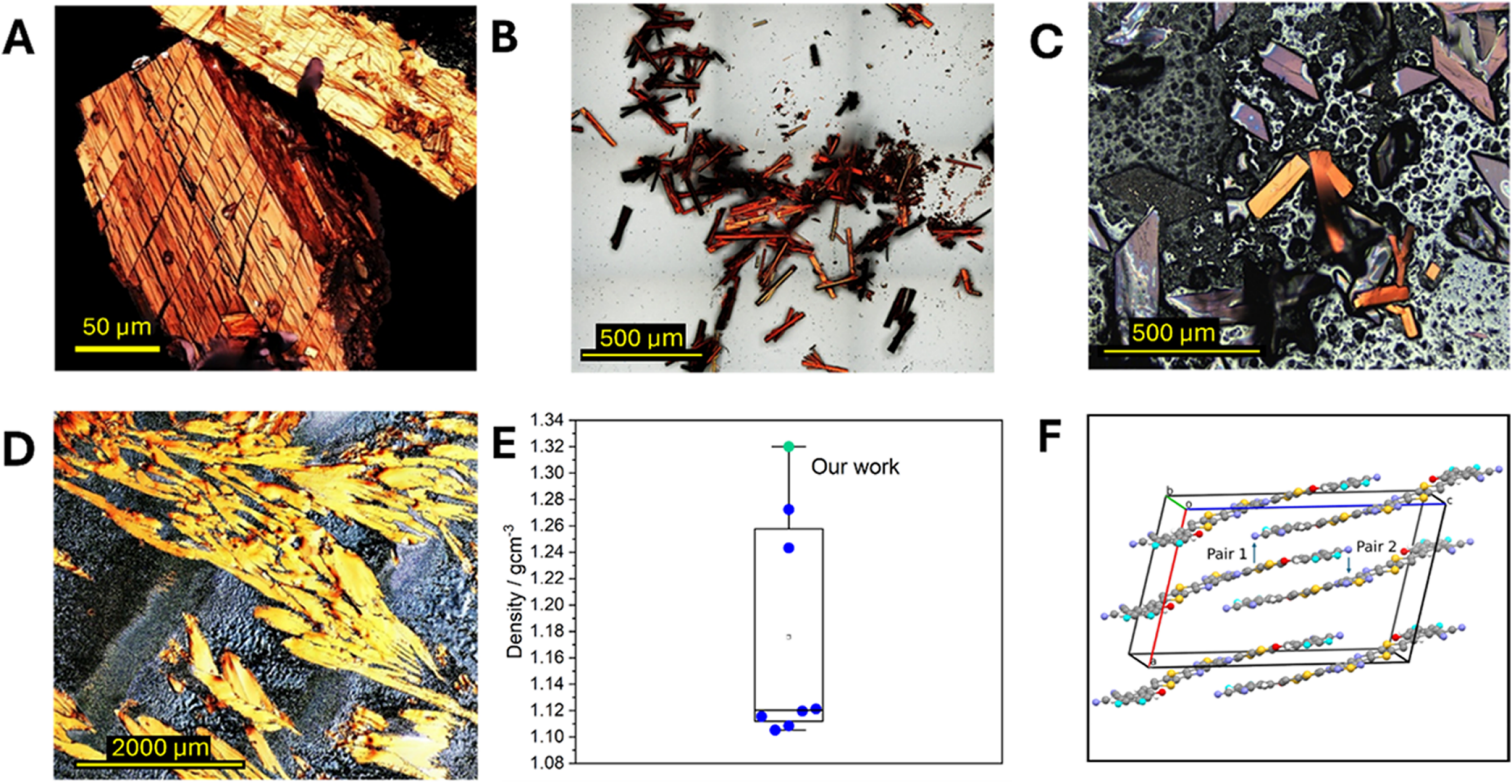
(a–d) Optical micrographs of Y6 crystal polymorphs, (a) metallic red crystals (MRCs)—50 μm scale (b) rod-like crystals—500 μm scale, (c) rhomboid-shaped purple, and rhomboid shaped metallic red crystals—500 μm scale, (d) spherulitic crystals—2000 μm scale (e) Density of the measured metallic red “cubic” crystals (green), compared to the literature values (blue dots—see Table 1 for references), (f) crystal structure of metallic red crystals with unique dimers highlighted.

Crystals with the same habits were observed when evaporating solutions from both phenol and acetophenone, which suggests the hydrogen bond donating ability of phenol may not be the key factor for the crystallization. All-atom molecular dynamics simulations have shown that as the π-conjugation degree of the additive increases, the strength of the additive-Y6 interactions increases, indicating this is the dominant mode of interaction.^22,32^ Although the solvation has not been explicitly modeled in this work, the same principles are assumed to apply.

Additionally, anisole was observed to dissolve Y6 well, but gave amorphous Y6 on solvent evaporation. According to the solvent classification system developed by Gu et al.,^33^ anisole belongs to the same group as the conventional halogenated solvents such as chloroform and chlorobenzene. In perovskites, chlorobenzene has been successfully replaced as an antisolvent by anisole due to their similar properties.^34^ Anisole is miscible in hexane,^35^ which further suggests it may similarly solubilize Y6 via both the aliphatic side chain and the backbone of Y6. As anisole has similar volatility to chlorobenzene, and is used in spin coating of PMMA thin films,^36^ it may be suitable for spin coating Y6 as a less hazardous alternative.

Crystals were also successfully grown from benzaldehyde, but longer evaporations led to oxidation of the benzaldehyde to benzoic acid, and the formation of blue benzoic acid crystals (i.e., Y6 in a benzoic acid matrix). As benzoic acid is much less volatile, Y6 could not be easily isolated from this matrix.

### 2.2 Crystal Structure and Comparison to Previous Polymorphs

Crystallization from phenol and acetophenone gave metallic red Y6 crystals, in contrast to both the blue-black color of the semicrystalline films and to most previously reported single crystals (and our own efforts at replicating the literature standard crystallization—which gave small dark red crystals of limited luster). Colors from previous reports are taken from the Cambridge Crystallographic Data Centre (CCDC) repository. While acetophenone gave crystals of similar habit and metallic red luster as those grown from phenol, the crystals were much smaller in size, hence we did not attempt to measure a crystal structure.

The crystal structure obtained from X-ray diffraction of the MRCs grown from phenol has notable differences to previously reported structures, detailed in Table 1. It is unique in that it is both the densest Y6 structure reported (Figure 2e) and the only structure where there is an axis of symmetry within the Y6 molecule (*Z*′ = 0.5), leading to a more symmetrical structure and only two unique sets of pairs (‘Y6 dimers’ in the single crystal—Figure 2f). Previously reported structures have at least one full Y6 molecule (generally 2 or 4) that is crystallographically distinct. Lower *Z*′, and denser structures, are both associated with more thermodynamically stable structures according to Kitaigorodskii’s principle of close packing.^37–40^ Exceptions to this rule largely occur in the presence of highly directional interactions, such as hydrogen bonding, which is less likely to apply to Y6. Detailed crystal packing diagrams are available in the Supporting Information Section 3.

**Table 1:** Crystallographic Descriptors of Y6 Polymorphs^a^

	1959113	2006203	2015912	1975854	2046756	2241856	2241854	This work (2531008)
Authors	Lin et al.^14^	Zhu et al.^15^	Zhang et al.^17^	Xiao et al.^12^	Zhu et al.^41^	Li et al.^16^	Li et al.^16^	
Year reported	2019	2020	2020	2020	2020	2023	2023	2026
Morphology	Prism	Needle	Needle	Needle	Block	Plate	Plate	Slab
Color	Black	Brown	Black	Black	Black	Black	Metallic dark black	Dark red
Crystal system	Monoclinic	Monoclinic	Triclinic	Triclinic	Triclinic	Monoclinic	Monoclinic	Monoclinic
Space group	*C*2/*c*	*P*2_1_/*c*	*P*1̅	*P*1̅	*P*1	*P*2_1_	*P*2_1_	*C*2/*c*
*a* [Å]	23.7019	15.1117	13.7272	14.4691	14.5264	15.1426	15.1269	17.0888
*b* [Å]	57.45	57.812	19.6561	21.1859	19.8017	57.4759	57.4352	16.0677
*c* [Å]	14.3969	20.076	29.7056	30.8424	28.5349	19.9312	19.8578	27.4389
α [deg]	90	90	102.45	109.352	95.143	90	90	90
β [deg]	118.541	95.923	92.677	96.264	101.463	94.85	94.584	103.805
γ [deg]	90	90	96.57	98.409	107.364	90	90	90
Volume [Å^3^]	17221.53	17445.52	7754.3	8697.73	7579.71	17284.7	17197.6	7316.48
*Z*′	1	2	2	2	2	4	4	0.5
*Z*	8	8	4	4	4	8	8	4
*D*_calc_ [g cm^–3^]	1.120	1.105	1.243	1.108	1.272	1.116	1.121	1.318
wR2, R1	0.5272, 0.1770	0.4581, 0.1624	0.4563, 0.1426	0.3136, 0.1117	0.4310, 0.1370	0.2788, 0.0848	0.1986, 0.0673	0.3031, 0.1143
*I*/σ(*I*)	3.5	9.7	4.5	8.8	13.1	14.7	17	21.6

a The top line shows CCDC numbers of reported Y6 crystal structures. *Z* is the number of Y6 molecules in the unit cell. *Z*′ is the number of Y6 molecules in the asymmetric unit. Where the modeled structure lacked the full alkyl chains, the density was corrected to account for the missing atoms. I/σ­(I) is a measure of the signal-to-noise ratio of the measurements, while lower wR2 and R1 indicate better agreement between the modelled structure and the raw data.

To understand better the origin of this increase in density, we decompose the density into an interplanar component (traditionally a “π–π stacking distance”) and a lateral density, i.e., packing density in the plane of the Y6 backbone. We note that this is not the same as the molecular plane, in particular due to the branched alkyl chains which are out of plane. This decomposition process is only meaningful for those polymorphs that are composed of “sheets” i.e., those with adjacent Y6 molecules in the same backbone plane. In this structure, the molecules are in exactly the same backbone plane, while crystal structures 2015912 (Zhang et al.) and 2046756 (Zhu et al.) (see Table 1), the two next densest structures, have Y6 molecules that are almost in the same plane. Hence these structures are chosen for comparison.

This analysis, summarized in Table 2, shows the origin of the higher density is a large increase in lateral density, outweighing a small decrease in interplanar density. More detail about this process is available in Supporting Information Section 3. The increased interlayer distance in the MRC structure leads to slightly greater interatomic distances in the dimers.

**Table 2:** Density Comparisons of Y6 Polymorphs Reported Here and in the Literature^a^

	This work (2531008)	2015912	2046756
Lateral density [molecules/Å^2^]	2.03 × 10^–3^	1.75 × 10^–3^	1.77 × 10^–3^
Vertical (“interplanar”) density [layers/Å]	0.269	0.291	0.299
Average interlayer distance [Å]	3.712	3.432	3.340
Total density product [g cm^–3^]	1.320	1.232	1.278
Total density product/CCDC density	1.00	0.99	1.00

a Total density products are calculated from the lateral densities (as measured using crystallographically equivalent atoms in the same plane) and vertical densities/interlayer distances (measured using crystallographically equivalent atoms in different planes). These are compared to the CCDC densities which are calculated using the number of molecules in the unit cell and its volume, demonstrating very close agreement.

### 2.3 Implications of Pair Analysis by DFT

To understand the photophysical effects of the newly identified crystal packing, we performed density functional theory calculations of exciton-charge transfer couplings.^27,41^ Due to its high symmetry, the MRC structure contains only two unique dimer configurations with π–π-stacking interactions, which we refer to as “Pair 1” and “Pair 2” (Figure 2f, and Supporting Information Figure S14). Pair 1 exhibits extensive cofacial contact between the Y6 chromophores, while π–π-stacking in Pair 2 is limited to the terminal subunits. Both structures exhibit close energetic alignment between the quasi-diabatic Frenkel exciton states (1.64 eV) and the intermolecular charge transfer states with the electron and hole localized on different molecules (1.54 eV in Pair 1 and 1.61 eV in Pair 2). This arrangement, with the emissive Frenkel exciton state thermally accessible from the charge transfer state, is consistent with the moderate, albeit lower, photoluminescence quantum yield for these samples compared to Y6 films. Based on comparisons of thick Y6 films and Y6 crystal photoluminescence signal strengths under the same excitation conditions, we estimate a steady-state PLQY in the crystals of ∼0.5–2%, though the uncertainty is large on this type of measurement.

As detailed in Supporting Information Section 4, despite the key differences in crystal structure between our MRC samples, and those crystals reported in the literature, the relatively similar FE-CT couplings suggest that the intrinsic photophysics and energetics of our crystals should be similar to those found in the literature samples. Though they are unusual for the general class of organic semiconductors, the FE versus CT levels are consistent with other theoretical^27,28^ and experimental^27,47^ observations of Y6 thin films (which suggest shorter singlet exciton lifetimes compared to total lifetimes measured by fluorescence lifetime spectroscopy). We observe a similar photophysical picture, as described in the sections below.

Pairs 1 and 2 are broadly reflective of the packing modes commonly seen in other Y6 crystal structures. The more extensive contact in Pair 1 is likely to be involved in exciton dissociation, due to greater stabilization of the charge transfer state as seen previously, while the “end on” π-stacking is commonly seen in a variety of fused-ring electron accepting materials.^27,28,42,43^

### 2.4 Morphology Characterization

To characterize the surface morphology of the Y6 crystals, we employed AFM in addition to optical microscopy. Larger metallic red crystals—of the type that XRD, and subsequent optical characterizations (detailed below) were performed on, showed various surface features—waviness, dislocations in the crystal surface, and ridges (Figure 3a). These features, and the softness of the crystals, reflect the fact that the crystal is composed of stacked sheets, with weaker intermolecular forces between them.

**Figure 3:**
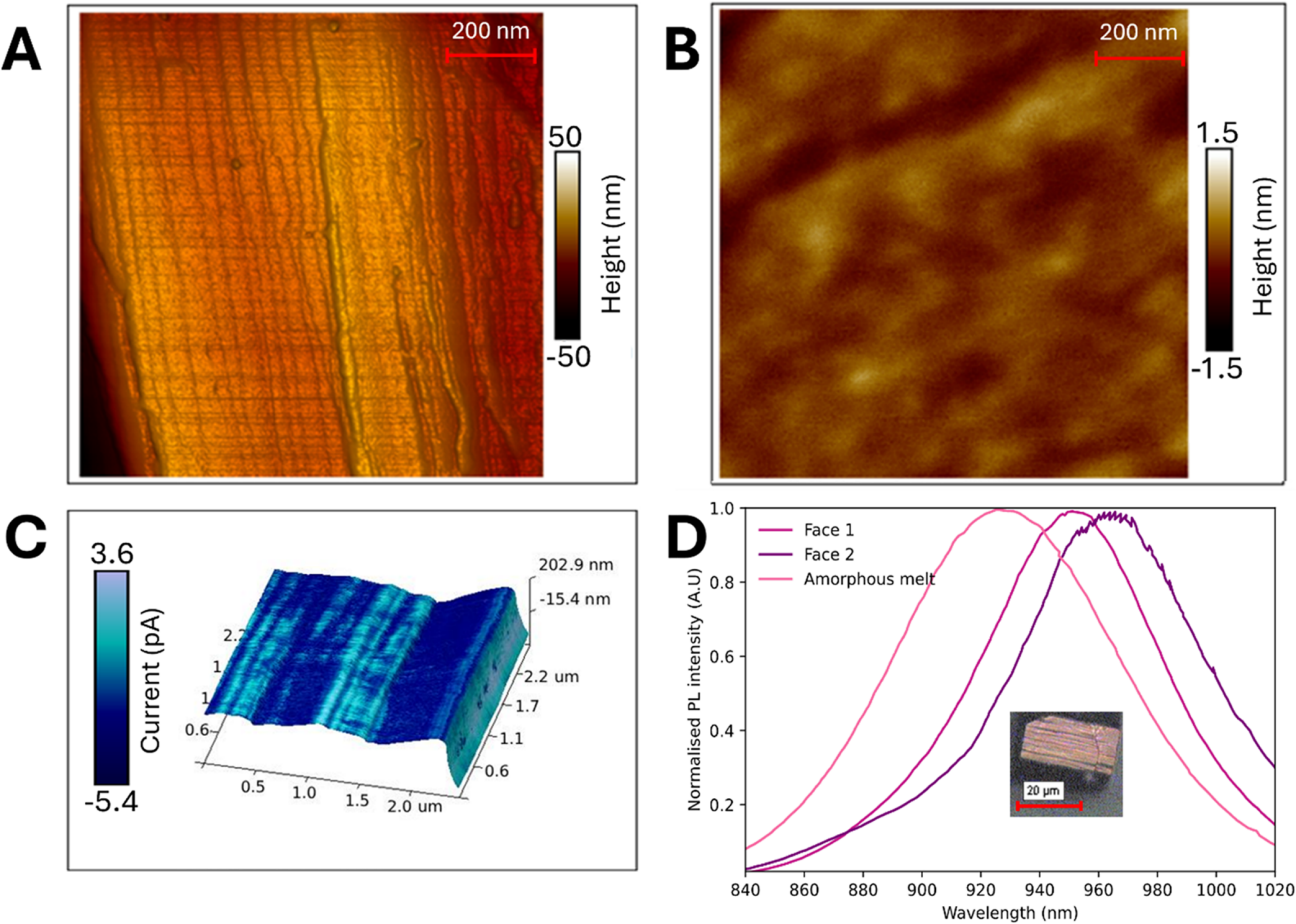
Surface morphology and roughness data from AFM measurements on (a) large metallic red crystals (roughness, *R*_q_ = 6.8 nm) and (b) thin platelike Y6 crystals (*R*_q_ = 0.2 nm). Ridges are observed at intervals of roughly 20 nm in the large cubic crystals. (c) 3D AFM topographic map of a crystal surface, overlaid with concurrent current signal as a blue colormap. (d) PL spectra from different spots on a single metallic red crystal compared to the spectra obtained from a semicrystalline/melted phase. Inset—microscope image of the measured crystal.

Thinner crystals (20–30 nm) were also investigated, as these have the most applicability to semitransparent organic solar cells because of their high transparency. These crystals showed a smoother surface over a larger area from the absence of the steps (*R*_q_, root-mean-square roughness = 0.2 nm), indicating a homogeneous surface and no grain boundaries (Figure 3b).

AFM conductivity data suggest that there are preferential charge transport channels in the MRCs at interlamellar boundaries. Single crystals of rubrene have shown highly anisotropic charge transport, with much higher hole mobility in crystallographic directions with π-stacking than without.^44^

### 2.5 Photophysical Properties of Y6 Crystals

As a nondestructive probe of the quality and novelty of the Y6 crystals, we performed a variety of steady-state and transient fluorescent spectroscopy measurements. Photoluminescence microscopy data from the MRCs showed sharper spectra, shifted into a longer wavelength region (Figure 3d), with a FWHM of the emission bands as low as 70 nm, compared to ∼90 nm for the semicrystalline/thin film phase. This spectral shifting and narrowing is consistent with expectations for a more “laterally dense” crystal structure, which may display more J-like, than H-like emission characteristics,^45^ and for a more crystalline film, which should show sharper and longer-wavelength emission due to enhanced singlet exciton-charge-transfer state hybridization.^28^ The changes in spectra for different faces of the crystal may also be due in part to optical effects such as modified reabsorption/waveguiding in the crystal.

Fluorescence lifetimes for several metallic red crystals were determined using fluorescence lifetime imaging microscopy (FLIM) and compared to those of a thick (>200 nm) Y6 film. Crystal fluorescence lifetimes were similar to the semicrystalline thin films (Figure 4a) and are provided in Supporting Information Tables 4 and 5. Both in thin films and the MRCs, the fluorescence decays showed multiexponential, or potentially nonlinear (nonexponential/power law) behavior. The multicomponent decays arise because of a variety of species present in photoexcited neat films, namely singlet excitons, charge**-**transfer excitons, triplet excitons, and potentially some polarons.^27,28,46,47^ For each sample, taking account of the instrument response function (IRF), the decays can be fitted with three exponential components: a fast, sub 100 ps feature, which is difficult to quantify accurately as it occurs on a similar time scale to the IRF (and can change in magnitude depending on different pump scattering effects); a roughly 1 ns decay component; and a longer lived ∼4 ns decay. Support for nonlinear effects contributing, even under the low pulse-energy pumping conditions of TCSPC, can be seen from the weak intensity dependence of the fast component (Supporting Information Figure S24) in crystal 1.

**Figure 4:**
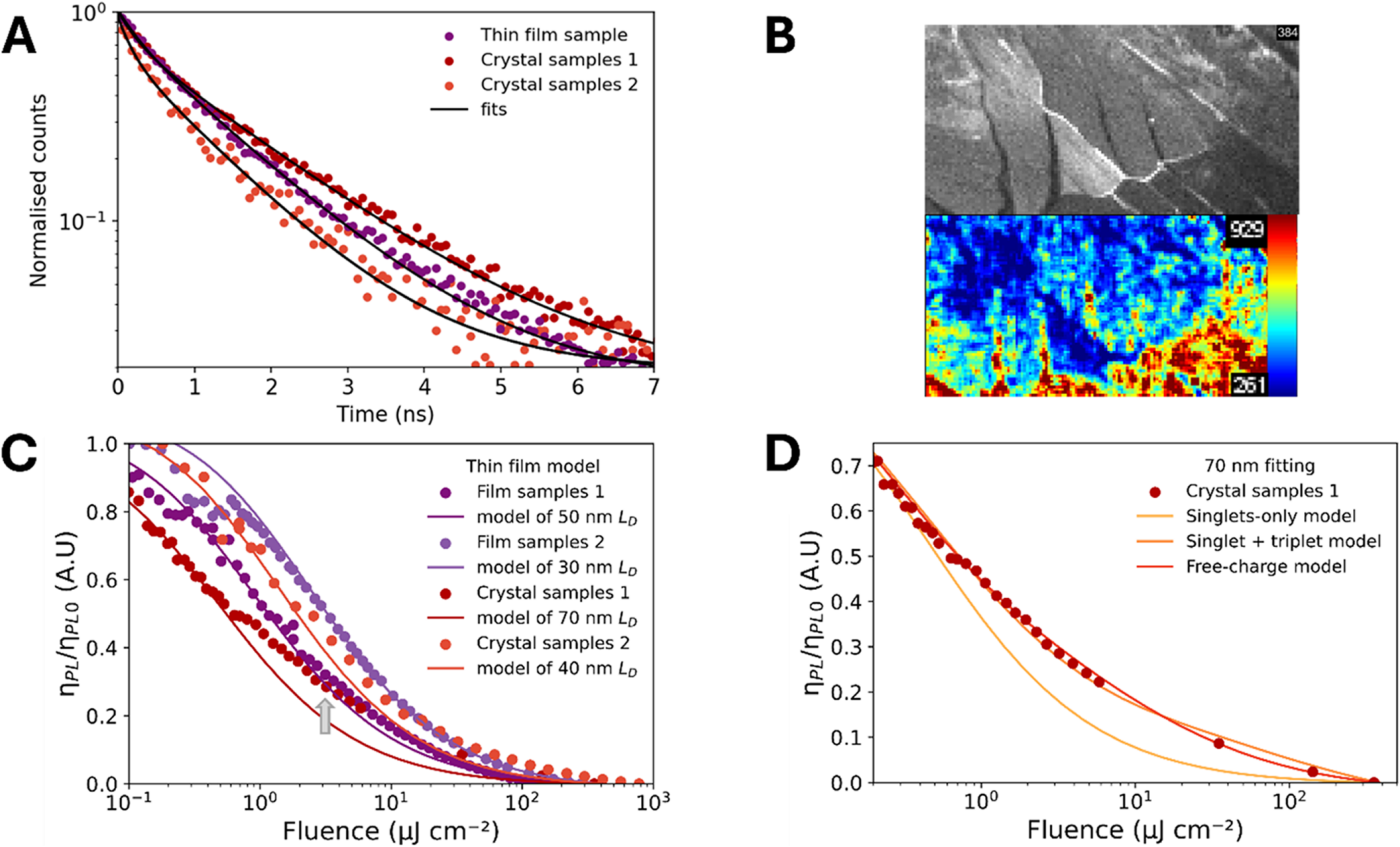
(a) Fluorescence lifetimes of crystal and film samples. Crystal samples 1 and 2 are densely packed ensemble metallic red crystals from different crystallization batches. Data were normalized at 320 ps to avoid prompt pump scatter signal. Black lines representing the best fit for the data, using a biexponential model, are shown for each of the samples, with weighted lifetimes of 1.07 (±0.04), 1.24 (±0.04), and 0.83 (±0.04) ns extracted for the film, crystal 1 and crystal 2 samples, respectively. (b) Top: FLIM image of Y6 aged metallic red crystals, bottom: FLIM image colored by lifetimes measured at each pixel. Large differences are observed in the ultrafast component due to pump scatter differences due to sample height. (c) Fluence-dependent pulsed PLQY measurements on film and crystal samples. Film samples 1 and 2 were made under the same conditions, with sample 2 having been exposed to air prior to measurement. Crystal samples 1 and 2 are the same samples described for Figure 4­(a) above (and in the main text). Samples were fit using the singlets-only model (see main text) to obtain diffusion lengths of 70 ± 15 nm for the crystal and 50 ± 10 nm for the film samples. A small arrow shows the high-fluence deviation observed in crystal samples compared to the model. (d) Y6 crystal sample data fitted to three different kinetic models.

Crystal fluorescence lifetimes varied across different batches of phenol-grown MRCs, but within individual crystalline domains, the lifetimes were constant—as shown in the color map of weighted lifetimes (Figure 4b). We present two representative data sets of crystal lifetimes alongside the film data in Figure 4a. The measurement labeled “Crystal samples 1” shows the fluorescence decay from a region of MRC domains from one batch of crystallizations on a watch glass. “Crystal samples 2” shows the fluorescence decay from the same size region of a different set of MRCs on a microscope slide. While “Crystal samples 1” showed a slightly larger average crystal domain size compared to “Crystal samples 2”, other factors, such as exposure to different humidity levels in ambient air during crystallization, may also account for the moderate differences in lifetimes.

We note that while the crystals measured via optical techniques described herein were grown using the same method as the discrete crystals analyzed by XRD, they are not the exact same crystals because the crystals which underwent X-ray diffraction showed degradation postmeasurement. Our analysis consistently resolves the same crystal structure for different metallic red crystals, as well as similar optical and crystal habits. We therefore attribute a consistent crystal structure to the metallic red crystals upon which we performed optical measurements. Crystal samples measured via nonmicroscopic fluorescence techniques represent an ensemble of these crystals.

To determine if the longer exciton lifetimes might translate to improved optoelectronic performance, and to interrogate the exciton diffusivity within the crystals, pulsed photoluminescence quantum yield (PPLQY) measurements were performed on both film and MRC samples using a method similar to Riley et al.^48^ The normalized intensity dependent photoluminescence yields (η_PL_/η_PL,0_) for the different samples are presented in Figure 4c. We compare these measurements to three kinetic models, described below. We note that for each model, care must be taken to estimate accurately the photoexcited carrier density within the film or near the surface of the crystal.

The first model (Model 1), shown as solid lines in Figure 4c, presents the simplest case for a purely excitonic population, with additional quenching of fluorescence arising solely from singlet exciton–exciton annihilation.^48^ This model applies even for strongly delocalized hybrid singlet–charge-transfer states, which the photoexcitations in Y6 thin films are known to be.^28^ This model shows an excellent fit to the thin film data, and a good match to the crystal data at lower fluences with some deviation at higher fluences (discussed further below). Exciton diffusion lengths, *L*_D_, are modeled by fitting the data to varying values of γτ—consisting of γ, the bimolecular exciton annihilation constant, and τ, the exciton lifetime.

Based on this analysis, we measure *L*_D_ values of 70 ± 15 nm for the crystal ensemble and 50 ± 10 nm for the film samples. We note that the greater diffusion lengths are observed despite the crystals being exposed to ambient air for a significant period during and after their crystallization. Exposing the Y6 thin films to ambient air results in substantial reduction of diffusion lengths (as shown in Figure 4c—where film sample 2 has been exposed to air, and film sample 1 has been kept and measured under inert conditions). We also see lower diffusion lengths for the crystal batches that showed reduced emission lifetimes. However, lifetime deviations extracted from FLIM measurements are too small to account for the changes in *L*_D_: the γτ values vary by a factor of 3, while lifetimes are within 15% of one another. The multiexponential decay seen in the FLIM data causes uncertainty in the precise lifetime values, but choosing a weighted average lifetime gives diffusivity estimates of (5.0 ± 1.2) × 10^–3^ cm^2^ s^–1^ for films, and (6.6 ± 1.6) × 10^–3^ cm^2^ s^–1^ for the best performing crystals. If we used the much shorter lifetimes quoted from the literature transient absorption spectroscopy data, then these diffusivity values would be even higher.

As mentioned above, at higher fluences (above ∼10 μJ/cm^2^), the metallic red crystal PPLQY data show a consistent deviation from expectations for the simple exciton annihilation case (Model 1). The higher-than-expected PLQY above these fluences could come from a specific thermal effect,^48^ but as we do not observe the associated spectral shifts, we instead attribute this relative increase in PLQY to enhanced triplet–triplet annihilation (TTA) which will generate additional radiative singlet excitons.

There are several plausible kinetic models that include TTA contributions. We present two additional models here (Model 2, and Model 3, shown in Figure 4d) as examples of improved fits to our PPLQY data while maintaining consistency with FLIM measurements. Details of these models are provided in Supporting Information Section 7. Briefly, in Model 2, in addition to an intersystem crossing term, we include a triplet generation term arising from singlet–singlet annihilation events. Model 3 is a more complex model used by Price, Hume et al.,^27^ that considers a portion of rapidly generated free charges, the recombination of which gives rise to a significant portion of triplet states which annihilate to match the PPLQY data.

For both models, and variations thereof, a substantial number of triplet state species, totaling a maximum of 25% total excited state population (population models shown in Supporting Information Figure S25), are required to achieve the measured relative PL enhancement at higher fluences. In Figure 4d the singlet exciton diffusion length is held constant at ∼70 nm for each model, and each shows a good fit to the data for fluences below 1 μJ/cm^2^. This constraint, and the good degree of fit to the low fluence data for multiple physical models, support the conclusion of record exciton diffusion lengths in the best performing Y6 crystals.

## 3 Discussion

The pulsed PLQY data reveal the novel photophysics of the Y6 crystals compared to their thin film counterparts. Higher exciton diffusivity values likely reflect more delocalized excitons enabled by greater crystalline order, rather than a simple reduction in defects (which would result in more significant lifetime changes than we observe). Increased triplet–triplet annihilation compared to films can be due to either generation of a larger number of triplet state species, or higher efficiency of the TTA process (or both). Greater delocalization of the singlet exciton would promote more triplet-state generation through each of the likely photophysical channels included in the kinetic models presented above: larger CT state proportions facilitate intersystem crossing between CT_1_ and CT_3_; enhanced singlet–singlet annihilation favors polaron generation, with polaron recombination giving rise to triplet state population.^49^ Finally, greater delocalization could also give rise to more directly generated free polarons which will also recombine into triplet state species. Due to the relatively similar PLQY’s of our metallic red crystals compared to thin films, and the behavior of our kinetic models to modification of the intersystem crossing rates (see Supporting Information Figure S18), we believe that the larger TTA contributions seen in the crystals are not likely to be caused by significantly different rates of intersystem crossing, but are most likely due to enhanced triplet exciton transport/lifetimes.

We note that singlet-polaron, and singlet–triplet annihilation may result in small reductions in the estimated *L*_D_ values. However, including these terms in our modified kinetic schemes and refitting our PPLQY data (Supporting Information Figure S18A) shows only a negligible decrease in estimated *L*_D_, due to the different lifetimes and fluence regimes where the singlet and triplet state species are dominant. Another unknown factor that may lead to the high-fluence deviations observed in Y6 crystals is the efficiency of singlet–singlet collisions that give rise to annihilation events—which has been shown to be more variable than is commonly assumed.^50^ In particular, theory suggests that exciton annihilation can be suppressed in H-aggregates, compared to J-aggregates, which may be relevant to our comparisons here.^51^

This work has important implications for devices reliant on nonfullerene acceptors, particularly for bilayer organic photovoltaics and light-emitting diodes.^8,52^ Employing phenol or acetophenone as more volatile but highly effective crystallization enhancing additives should provide a way to enhance the efficiencies of both OPVs and OLEDs. While it is difficult to disentangle the effects of different polymorphs versus improved crystallinity, the observed differences in photophysical pathways and lifetimes between our crystals and thin films highlight the developing understanding of the importance of crystal packing on optoelectronic properties,^49,53,54^ not just for charge transport but for excitonic properties as well.^7^

The greater lateral packing density and structural order of the crystals might contribute to their higher performance despite exposure to ambient conditions, potentially offering a useful design rule for enhancing stability in organic photovoltaic devices. However, we note that initial estimates of laser-induced photodegradation in the presence of air show a similar drop in fluorescence intensity with time for both crystals and thin films (Supporting Information Figure S23).

Particularly promising is the fact that the crystallization technique described here is generalizable to other nonfullerene acceptor compounds. We show large and high-quality crystals of the nonfullerene acceptor IDIC in Supporting Information Figure S2. This new approach opens the door to exploring a range of large and high purity crystal polymorphs of a series of nonfullerene acceptors, which in turn may give rise to exciting new device prospects. An area of particular interest for future investigation could be the potential for making photonic devices from large nonfullerene acceptor single crystals, given the success of nonfullerene LEDs,^8^ and hybrid perovskite single crystal lasers.^55^

## 4 Conclusion

We report a new crystallization technique that reduces the required time to crystallize nonfullerene organic semiconductors to a matter of hours. The technique provides large crystals of Y6 with a structure that is denser than previously reported. These crystals show novel photophysical responses not seen in thin-film samples, and record exciton diffusion lengths. These findings open up new avenues for nonfullerene acceptor crystal research by enabling high quality optical and X-ray probes of crucial material properties, advancing their potential for use in high-performing optoelectronic devices.

## 5 Experimental Section

Y6 was purchased from Aaron Chemicals. Phenol, anisole, acetophenone, and benzaldehyde were purchased from Sigma-Aldrich. All chemicals were used without further purification.

Y6 was dissolved in a solvent selected from phenol, anisole, acetophenone and benzaldehyde, at concentrations from 0.1 to 5 mg mL^–1^. The solvent was allowed to evaporate from a watch glass at a low oven temperature (50–70 °C). In general, lower oven temperatures and less concentrated solutions gave better conversions to crystals, but smaller crystalline domains.

Y6 crystals were grown from acetophenone by convection. The principle of this (re)­crystallization method is that there is a heat gradient in an angled container; at the cold top of the vessel, the crystals nucleate and begin to grow.

X-ray crystallography^56^ data were collected on a Rigaku 007HF diffractometer with HF Varimax confocal mirrors, an UG2 goniometer and HyPix Arc-100 detector. The crystal was kept at a steady *T* = 100(2) K during data collection. The structure was solved with the ShelXT (Sheldrick, 2015)^57^ solution program using dual methods and by using Olex2 1.5 (Dolomanov et al., 2009)^58^ as the graphical interface. The model was refined with ShelXL 2018/3 (Sheldrick, 2015)^59^ using full matrix least-squares minimization on F2.

The crystals of Y6 were composed of stacked sheets, evidenced by diffraction pattern smearing in the stacked direction depending on crystal thickness. Care was taken to measure crystals before radiation damage became too severe. The stacked crystal was modeled as a single crystal with a nonmerohedral twin law applied to the integrated data set.

CCDC2531008 contains supplementary X-ray crystallographic data for “Y6”. These data can be obtained free of charge from the Cambridge Crystallographic Data Centre via www.ccdc.cam.ac.uk/data_request/cif.

Time-dependent density functional theory calculations were performed in Gaussian 16,^60^ using the Tamm-Dankoff approximation, the CAM-B3LYP^61^ exchange-correlation functional and a 6-31+(d,p) basis set. Dielectric stabilization was treated using a polarizable continuum model with a low frequency dielectric constant of 3.8.^62,63^ the exchange–correlation functional was range-tuned according to the established nonempirical procedure,^43,64–66^ resulting in an optimal range-separation parameter of 0.0143 Bohr^–1^. Electronic coupling calculations were conducted on pairs of molecules extracted from the X-ray crystal structure, with alkyl chains truncated to methyl. Electronic couplings and exciton/CT energies were accessed by performing calculations in which the molecules were separated from one another by ∼10 Å to define localized CT states, and pure exciton states that were then localized by diabatization.^67^ Finally, the energies and couplings between these states were calculated by projection onto the states of the molecular pair in the crystal packing geometry.^41,68^

Steady-state microphotoluminescence measurements were carried out on a Renishaw 1000 Raman system using a 1200 l/mm grating, coupled to a Leica DMLM optical microscope. Excitation was from a 514.5 nm diode laser.

Optical microscopy measurements were carried out using a LEXT OLS5100.

Atomic Force Microscopy (AFM) was conducted in ambient environment using a Multimode VIII microscope [Bruker, CA, USA] with Nanoscope V controller with PeakForce feedback control and PeakForce Tunneling AFM (TUNA) module as well as a Nanowizard V AFM in tapping mode as part of the University of Bristol AFM Technology Platform. For PeakForce topographic imaging SCANASYST-AIR-HR cantilevers of nominal tip radius and spring constant 2 nm and 0.4 N/m were employed with < nN force control. PeakForce TUNA conductivity mapping was conducted using PFTUNA cantilevers of nominal tip radius 25 nm and spring constant 0.4 N/m, with < nN force control and 1–8 V applied sample bias on crystals mounted on conductive copper tape. Topographic mapping on the Nanowizard V employed SCOUT 350 RAu [NuNano, Bristol, UK] of nominal tip radius <10 nm and spring constant 42 N/m.

Ellipsometry was carried out using an Accurion ep4 imaging ellipsometer operating in RCE mode. Measurements were taken from 450 to 1000 nm using a 10× objective which allowed individual crystals to be investigated. The real (*n*) and imaginary (*k*) components of the refractive index were modeled from measured values of δ and ψ using a combination of Tauc-Lorentz and Gaussian oscillators.

PLQY of metallic red crystals samples was estimated by first measuring the PL counts of a ∼200 nm thick film of spin-coated Y6, excited at low intensity with 800 nm light, and then placing the MRC samples in the same sample configuration and position as the thick Y6 film, and comparing the PL counts between the two samples. The PLQY was then estimated by multiplying the reported literature PLQY values for Y6 films by the ratio of our measured PL counts for the two samples.^27^

Excitation pulses of 800 nm light were generated by an amplified titanium sapphire laser (Coherent Vitara-S oscillator and Legend Elite+ amplifier, 4 W output power) with a repetition rate of 1 kHz and a pulse duration of 35 fs. The laser light was attenuated by neutral-density filters before being directed toward the sample and focused using an off-axis parabolic mirror to a spot size of several hundred μm, as measured using a GenTech beamage-4 M beam profiler. The PL was collected using another off-axis parabolic mirror, and focused into a Shamrock 303i spectrometer with a Newton CCD detector attached. Pump powers at the sample were measured using a Thorlabs S121C standard photodiode power sensor.

Fluorescence lifetime images were acquired on a LEICA TCS SP8 system attached to a Leica DMi8 inverted microscope (Leica Microsystems). Excitation was provided by a white light laser (NKT Photonics SuperK Extreme, packaged as WLL/WLL2) with a repetition rate of 80 MHz and pulse duration of 5 ps. An acousto-optical beam splitter (AOBS) selected an excitation wavelength of 561 nm. Fluorescence was detected using a hybrid detector operating in photon counting mode over an emission range of 616–795 nm. A notch filter centered on 561 nm minimized any laser scatter into the detector. Time resolved data were acquired using a PicoHarp 300 TCSPC module (PicoQuant) controlled through SymPhoTime64 software (PicoQuant). Fitting of FLIM images was performed with the FLIMfit software tool (version 5.1.1) developed at Imperial College London.^69^ Tail fitting of the fluorescence images was performed pixel-wise allowing spatial variations in the fluorescence lifetime to be visualized.

## Supplementary Material



## Data Availability

Raw data, images, and analysis code are available at the repository https://github.com/Thomas-Romain/Non-fullerene_crystallization_python_code

## Notes

Information on additional measurements and descriptive methods of modeling performed (Figures S1–S23) (Tables S1–S6) (PDF)

## Abbreviations

NFA: nonfullerene acceptors
OPV: organic photovoltaic
PCE: power conversion efficiency
LED: light-emitting diode
OFET: organic field effect transistor
HBD: hydrogen-bond donor
AFM: atomic force microscopy
PLQY: photoluminescence quantum yield
PMMA: poly methyl methacrylate
CCDC: Cambridge Crystallographic Data Centre
DFT: density functional theory
FE: Frenkel exciton
CT: charge-transfer
FLIM: fluorescence lifetime imaging microscopy
FWHM: full width at half-maximum
IRF: instrument response function
TCSPC: time-correlated single photon counting
TTA: triplet–triplet annihilation
