# Supplementary material for: Record Carrier Diffusion Lengths in Large, Dense Nonfullerene Electron Acceptor Crystals Grown from Polar Aromatic Solvents
Source: J Am Chem Soc. 2026 Jul 6;148(27):28881–92. doi: 10.1021/jacs.6c06671 (PMC13383712; doi:10.1021/jacs.6c06671)
Supplement: Supplementary file 1 [file ja6c06671_si_001.pdf]

Supporting Information

Record carrier diffusion lengths in large, dense non-fullerene electron acceptor crystals grown from polar aromatic solvents

Tamir Halevi[†‡], *Thomas A.L. Romain*[†‡], *Paul A. Hume*[∇], *Peter N. Horton*[§], *Simon J. Coles*[§], *Robert L. Harniman*[†], *Ava Blandford*[†], *James A. Smith*[†], *Richard Cousins*[‖], *Dominic Alibhai*[Δ], *Andrew J. Orr-Ewing*[†], *Simon R. Hall*[†]*, *Michael B. Price*[†]*

† School of Chemistry, University of Bristol, Bristol, BS8 1TS, United Kingdom

∇ School of Chemical and Physical Sciences, University of Wellington, Wellington, 6012, New Zealand

§ National Crystallography Service, University of Southampton, Southampton, SO17 1BJ, United Kingdom

Δ Wolfson Bioimaging Facility, University of Bristol, Bristol BS8 1TD, United Kingdom

‖ Nanoscale and Microscale Research Centre, University of Nottingham, Nottingham, NG7 2RD, United Kingdom

Section 1: Liesegang ring images 3
Section 2: Y6 crystal polymorphs 4
Section 2B: Concomitant polymorphism 7
Section 3: Detailed crystallographic analysis 10
Section 4: DFT calculations 18
Section 5: Micro-ellipsometry 21
Section 6: Calculation of photo-excited carrier densities 21
Section 7: Kinetic models of pulsed PLQY 23
Section 8: Error analysis and kinetic model bounds 28
Section 9: Thermal and photostability measurements 30
Section 10: Solvent volatility analysis 37
References 38

Section 1: Liesegang ring images

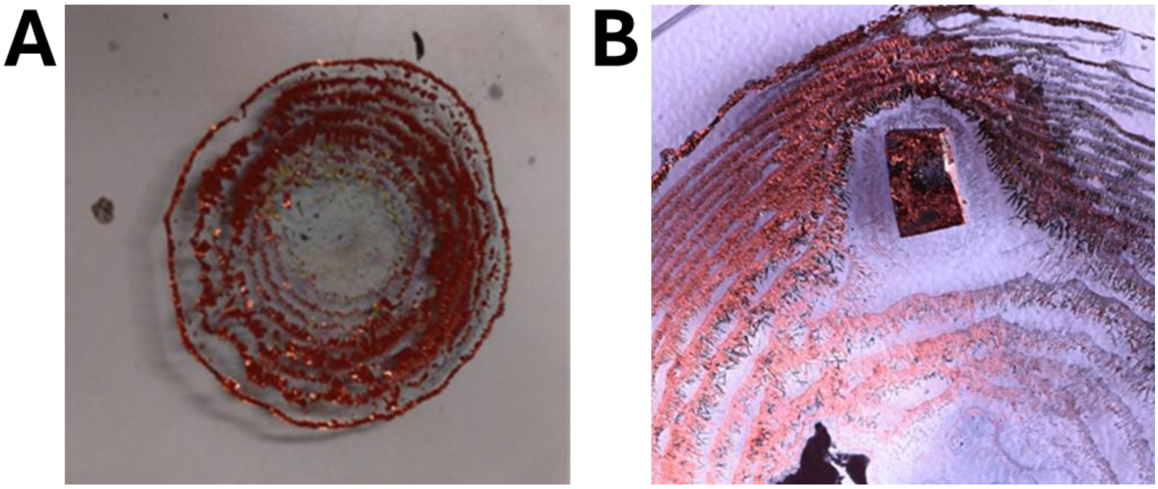

Figure S1. Liesegang rings of A) IDIC crystals and B) Y6 crystals, grown on watch glasses from phenol, as described in section 2.1 of the main text. In both cases the largest crystals are present on the outer edges, with the smallest crystals in the centre of the watch glass being interspersed with uncrystallized amorphous material.

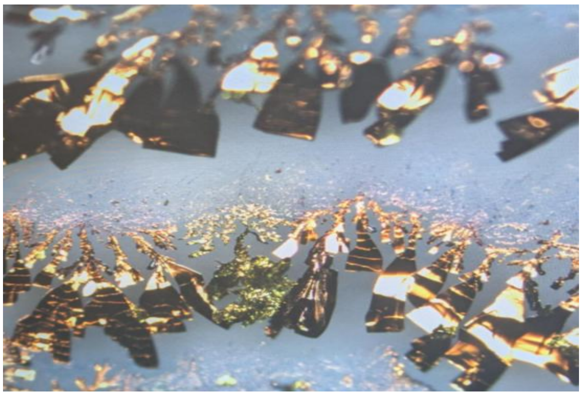

Figure S2. Magnified image of the IDIC crystal rings from Figure S1 showing post-nucleation Ostwald ripening.

Section 2: Y6 crystal polymorphs

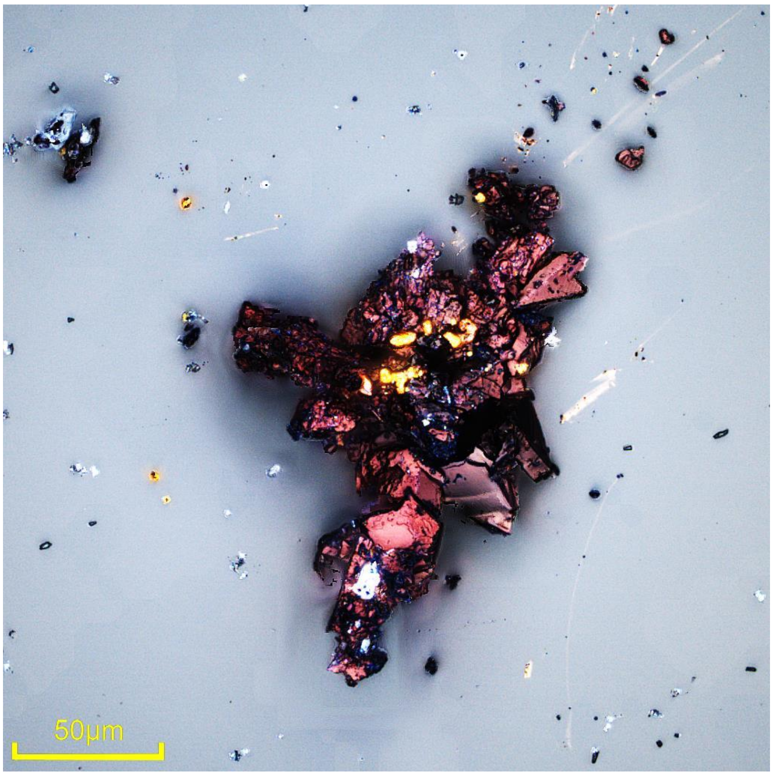

Figure S3. Y6 crystals grown by antisolvent diffusion of acetone into chloroform, through an intermediate layer of dichloromethane. Crystals show similarity to those grown from non-traditional solvents in the observed colours and metallic sheen, and differ in the high degree of polycrystallinity

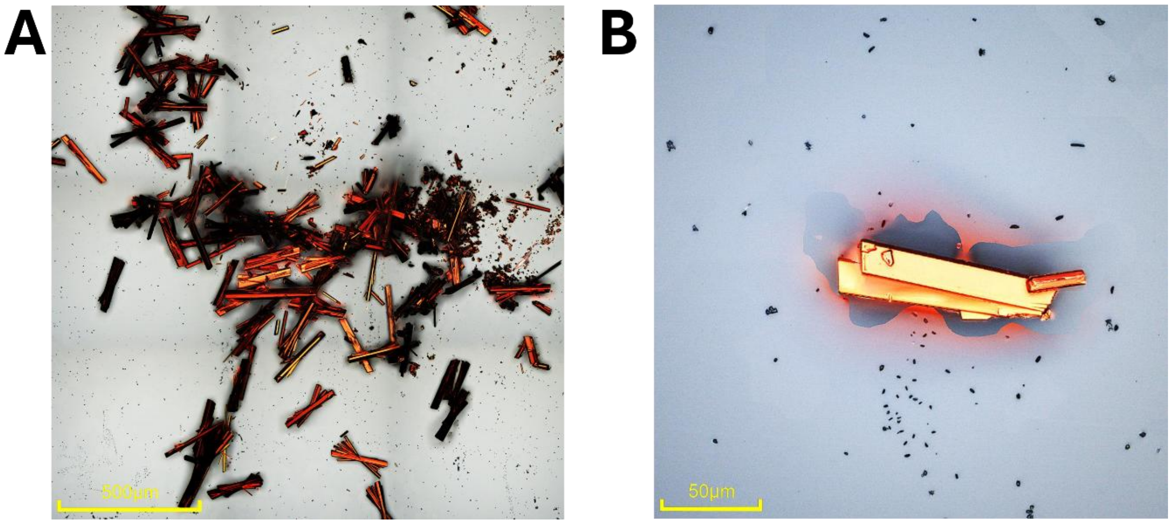

Figure S4. Crystals of Y6 grown via convection in acetophenone, as described in section 2.1 of the main text, with A) multiple crystals shown in a large cluster, up to 300 μm in length and B) a zoomed micrograph of a single crystal, around 100 μm in length.

Y6 crystals were grown from acetophenone by convection. The principle of this (re)crystallization method is that there is a heat gradient in an angled container; at the cold top of the vessel, the crystals nucleate and begin to grow.

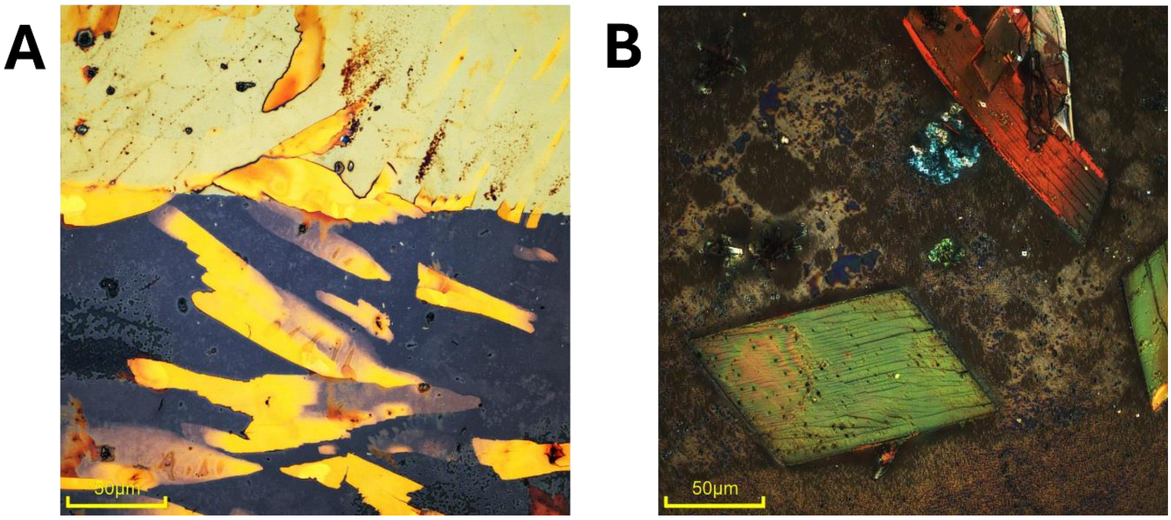

Figure S5. Crystals of Y6 grown from phenol on substrates of A) ITO B) silicon. Substrates were placed in watch glasses and the crystallization proceeded as described in section 2.1 of the main text.

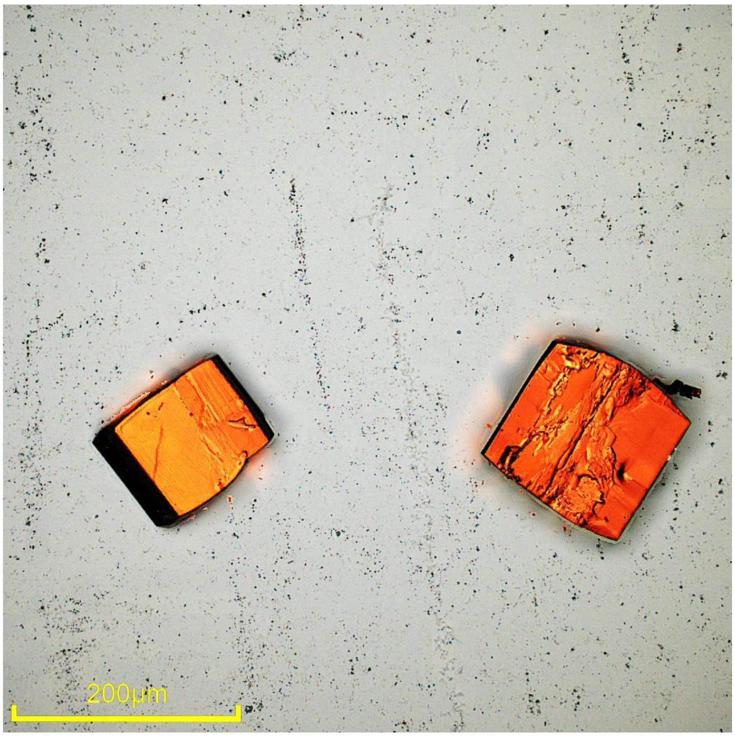

Figure S6. Crystals of Y6 grown by seeded growth from acetophenone.

Crystals were grown by seeding using a cubic crystal. A saturated solution was prepared at 60 °C by adding excess Y6 to acetophenone and, after allowing time to fully dissolve, filtering the excess and adding a cubic seed crystal. The solution was then allowed to cool slowly. The resulting crystals also appeared cubic, which suggests that the rod-like and cubic crystals are different polymorphs. The seeding experiment was conducted at the same supersaturation (or lower, as the solution was filtered), and therefore growth rates of different faces of the same polymorph should be similar.

Section 2B: Concomitant polymorphism

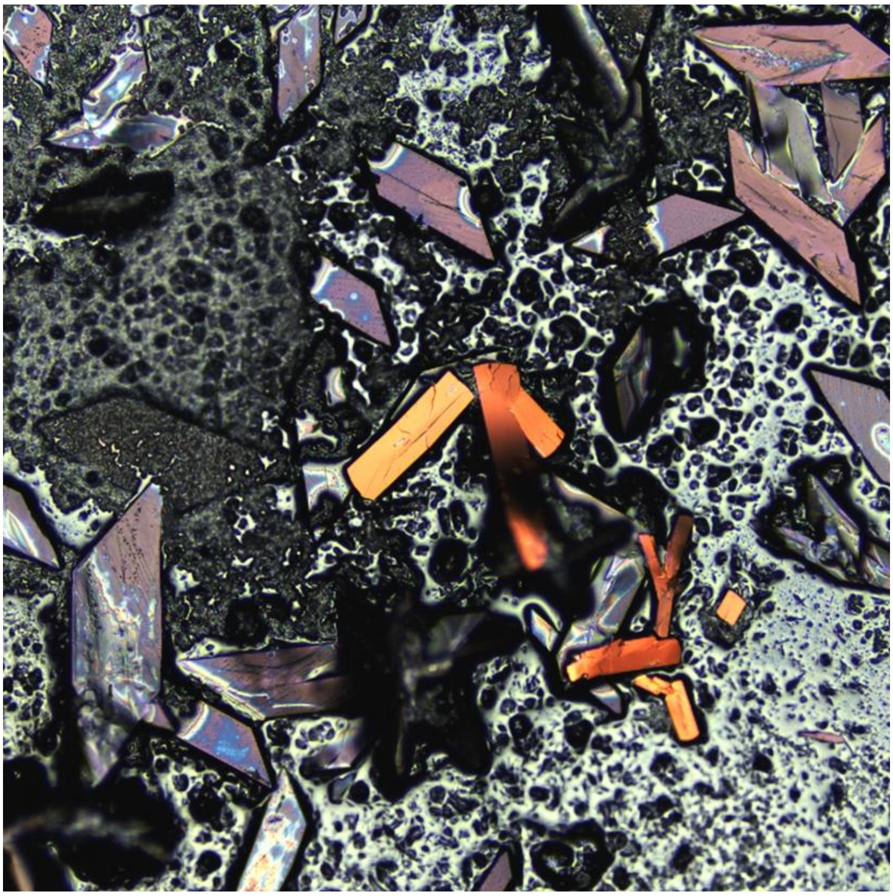

Figure S7. Optical micrograph of plastic substrate after evaporation of Y6 in phenol solution

When crystals were grown on a plastic substrate, with phenol as a solvent, two distinct crystal phases were observed: orthorhombic purple crystals, and rectangular / rodlike orange crystals (**Figure S7**).

The concomitant growth of these polymorphs implies that they have similar thermodynamic energies. Previous reports of concomitant polymorphism have included the observation of changes in colour resulting from minor changes in packing structures.1 In rigid π-stacked systems, colour polymorphism (or crystallochromy) has been suggested to originate from slight differences in packing.2 The polymorphism occurs when the dominant packing interactions (e.g. π-stacking) are similar, but the interstack packing is different. As the dominant interaction is similar, the overall energy is similar, leading to comparable growth rates.

In a related, more unusual phenomenon, crystals with striations of different colours were observed (Figure S8).

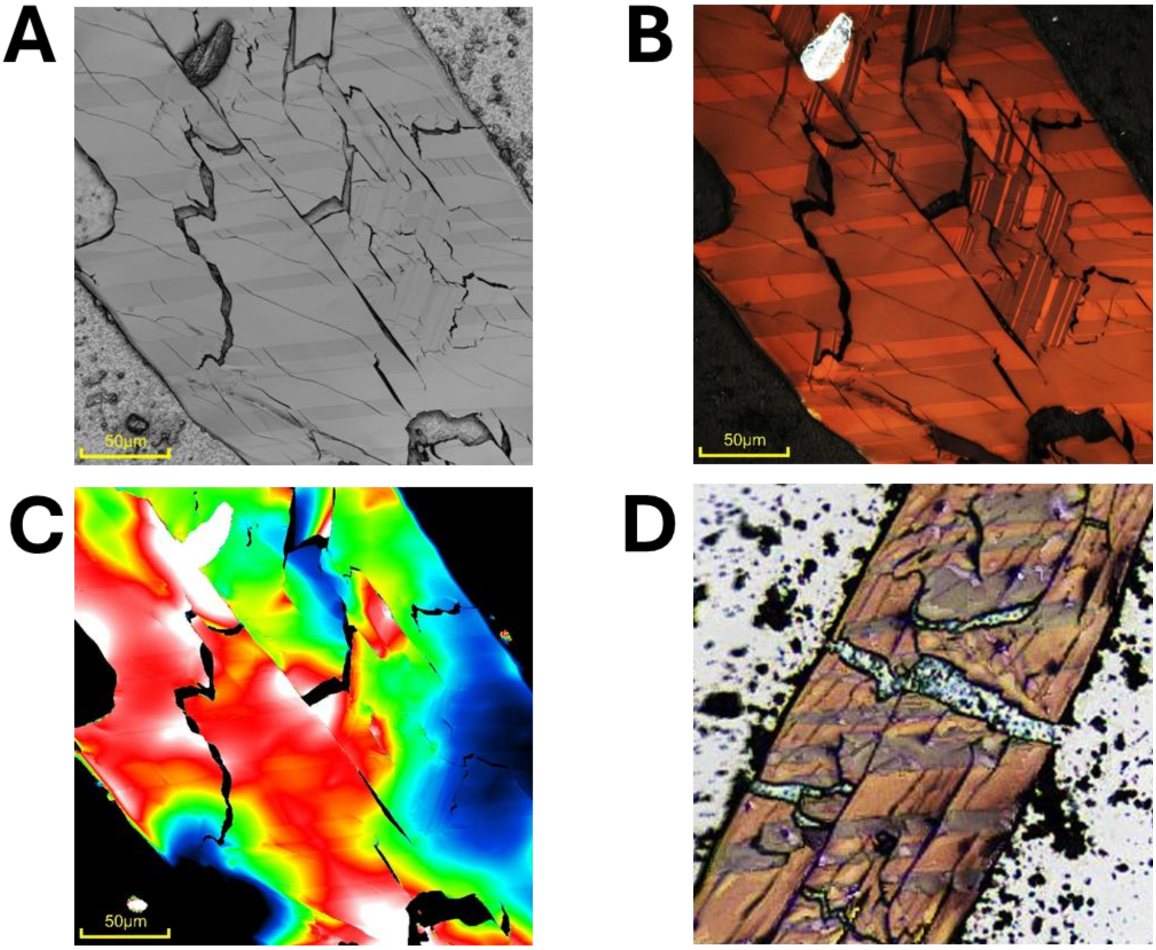

Figure S8. Optical micrographs of striated Y6 crystals. Images A-C in **Figure S8** were taken at the same position on the crystal. A) Laser intensity image, allowing the glass substrate and

amorphous crystalline to be seen more clearly. B) Polarized white light image. C) Height data of the crystal. Micrographs A to C show small fringes present in the crystal; while the fringes are visible in the height data, the colour of the region visible in B) does not correlate with height. D) a different striated crystal under unpolarized white light

Polysynthetic twinning (where twin crystals intergrow in parallel) is known in certain minerals, such as albite, but these tend to result in very minor parallel lines, rather than a change in colour.[3] Cross-seeding is a related (but distinct) phenomenon where crystals of one polymorph act as seeds for another.[4] Without observation of the crystal growth, it is difficult to know whether one polymorph grew first, and seeded the other polymorph. However, the alternating pattern with roughly parallel boundaries seems unlikely to have formed separately (with the second polymorph filling the gaps)—rather, as the crystal grew, the polymorph repeatedly changed. If this is understood as cross seeding, it implies that both polymorphs are able to seed one another, which has not previously been reported for organic semiconductors.[5] However, intergrowth of aspirin polymorphs has been reported, and was rationalized by the understanding that transforming one polymorph into another involves the breaking and reforming of weak bonds.[2] This rationale is consistent with the empirical observation that the crystals are soft and easily smeared, and the observation from the single-crystal structure that they are composed of stacked sheets. In the case of aspirin, the polymorphs are almost identical but involve slight differences in CH· · · O bonding – it seems likely that the blue and orange polymorphs differ primarily in weak bonding interactions, leading to the difference in colour. Slight height differences were observed between each region suggesting discontinuity between the regions and indicating they are indeed different crystallographic domains. Although such intergrowth of two polymorphs with different colours has not been previously reported, we consider it the most likely explanation for the observed striations shown in **Figure S8**.

Section 3: Detailed crystallographic analysis

As mentioned in section 2.2 of the main text, due to lateral offsets in dimers, interplanar distances can be misleading as they may not reflect the real distance between molecules. Additionally, since the molecular backbone is not perfectly planar, parts of the backbone that lie above or below the defined plane will have different distances to the other molecules. For example, in our reported metallic red crystal (MRC) structure the N4C1 distance is just 3.36 Å because the atoms involved are both out of plane towards each other (**Figure S9**).

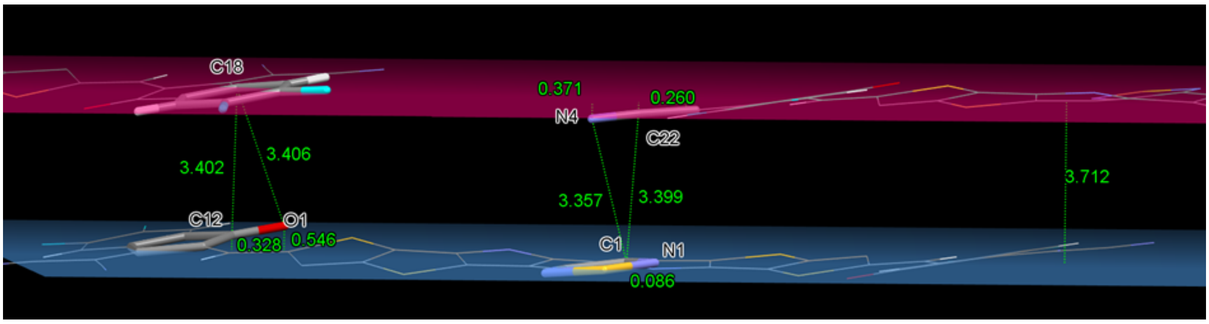

Figure S9. Selected intermolecular distances in the MRC structure that are significantly closer than the interplanar distance.

The planarity of the MRC structure was therefore evaluated in comparison to these two polymorphs.

Table 1: Planarity of the molecules in Y6 polymorphs.

Structure		Mean distance atom-plane, S.D. [Å]	Median distance atom-plane [Å]	Maximum distance atom-plane [Å]	Twist angle [º]
2046756	A	0.19, 0.15	0.15	0.74	11.41
	B	0.17, 0.16	0.11	0.62	14.82
2015912	A	0.19, 0.11	0.18	0.47	12.07
	B	0.14,0.10	0.12	0.41	10.56
Metallic red		0.19, 0.14	0.16	0.55	4.81

The planarity refers only to atoms of the molecular backbone. A and B are crystallographically distinct molecules in the crystal structures. The twist angles between the terminal units (the outer fused rings) are provided to further demonstrate how parts of the Y6 molecules may twist out of plane affecting intermolecular/interatomic distances. We approximate an error of 0.03 A for the associated atom-plane distances based on the atomic displacement parameters, and stress the high resolution of data minimises associated uncertainties sufficiently to enable fair estimations of how planar the molecules are.

The analysis demonstrates firstly that the structures are all quite planar, as expected from a conjugated ring system – the averages are not significantly different from each other, although 2046756 (L. Zhu et al.) appears to have slightly higher extremes, reflecting (for example) a high twist angle for one of its malononitrile groups in the A molecule. Thus, it can be assumed that from their closer average interplanar starting points, the other two structures will also show distances that are closer due to out-of-plane atoms. Without exhaustively measuring all the interatomic distances, this is corroborated by the fact that 2015912 (G. Zhang et al.) shows ~50 interatomic interactions <3.5 Å compared to ~30 for the metallic red (per Y6 molecule). It is true that although 2015912 has an interlayer spacing 0.28 Å closer than the MRC structure, the closest interatomic distance is 3.21 Å (just 0.15 Å closer). Furthermore, there are just 5 interatomic distances more than 5% closer than the interplanar distance per Y6 molecule, in comparison to 52 for the MRC structure.

Therefore, π-π stacking distances, or interplanar stacking distances, are not the whole picture, and do not account for offsets or deviations from planarity, especially if the atoms are out-of-plane towards each other. This analysis means that the interatomic distances are more similar in these structures than the interplanar distances suggest.

Overall, however, the interplanar distance is still predictive of closer interatomic contacts and presumably stronger orbital overlap.

A
B
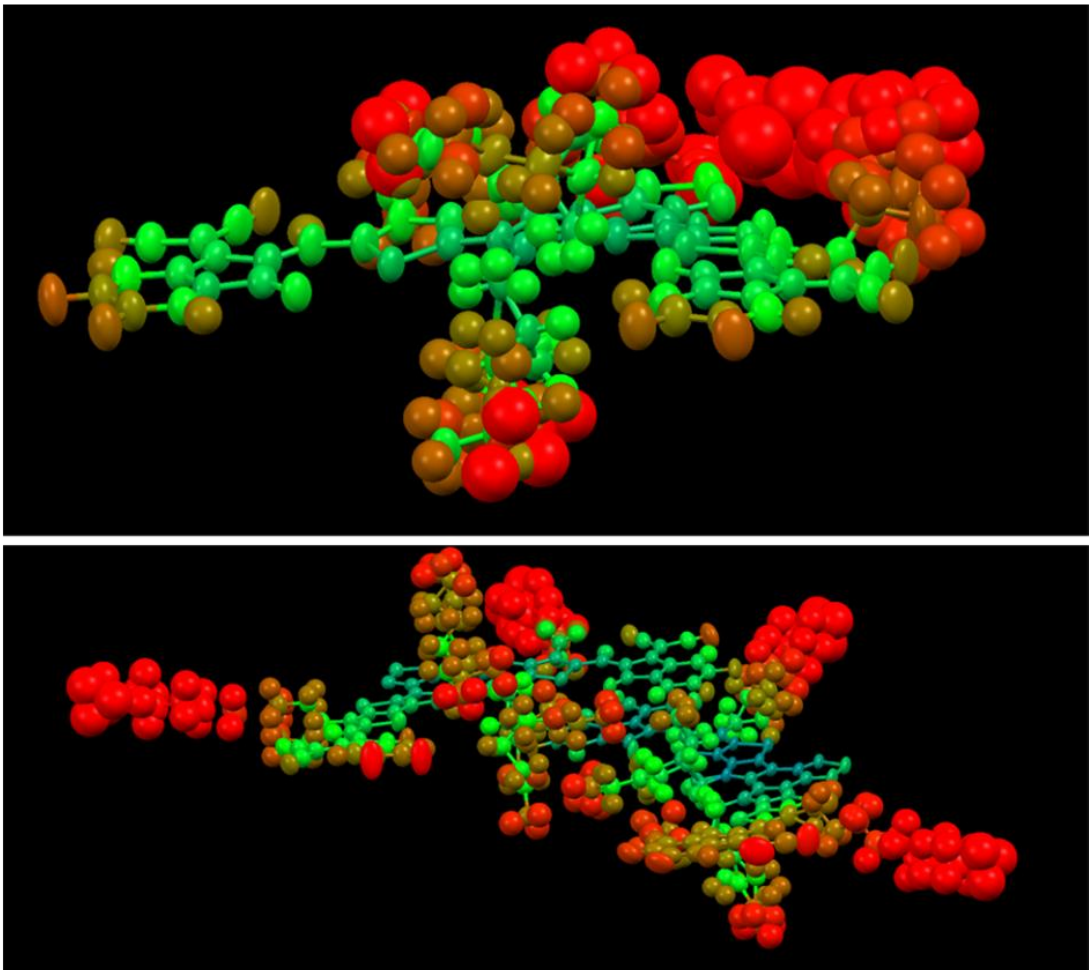

Figure S10. An ellipsoid view of A) metallic red and B) 2046756 crystal structures. The atoms are coloured by their isotropic equivalent atomic displacement parameter (redder = higher), and the ellipsoids show the area in which there is a 50% probability of finding the electron density associated with an atom. Note the branched alkyl chains, highlighted with blue arrows, have more atoms with lower displacement parameters.

The higher I/σ(I) value, representing the signal to noise of the X-ray diffraction measurement, is indicative of the better quality of the data attributed to the larger size and lower disorder in the metallic red crystals. We were able to explicitly model the complete alkyl chains, fully solving the structure, where previously the alkyl chains had been only partially modelled (if at all). The disorder is modelled across two distinct sets of positions for the alkyl chains, which

is a compromise between representing a continuum of positions and overfitting noise. The structure does show a HH distance that is too short to be physical— this is reflective of the disorder in the structure. The short distance is accounted for by the modelling, as it is between a low occupancy position (0.328) and a high occupancy position (0.6). Therefore, the model does not require or assume that both positions are occupied at once. The atoms in the linear alkyl chain have an average (isotropic equivalent) atomic displacement parameter of 0.197 $Å^2$, compared to 0.125 $Å^2$ for the branched alkyl chain. This means there is more uncertainty in the positions of the linear alkyl chain atoms, and is to be expected, due to the more flexible nature of the linear alkyl chain.

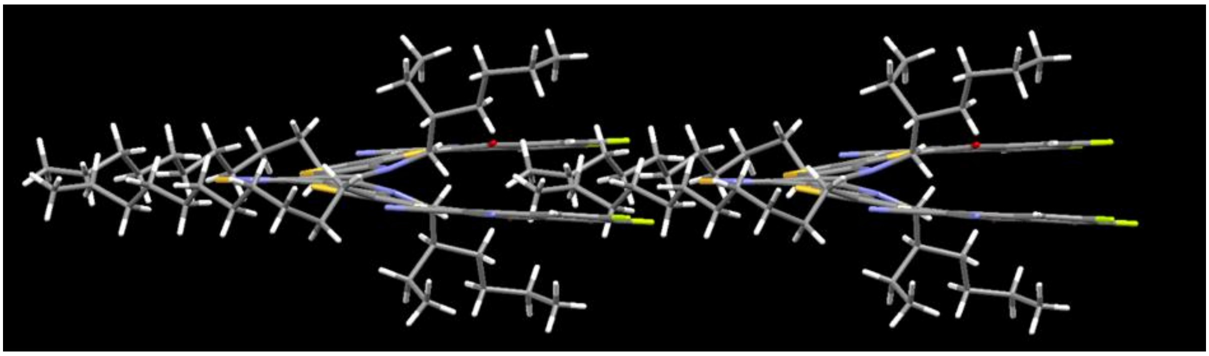

Figure S11. Overlap between the linear and branched alkyl chains of molecules in the same column. For clarity, only the major disordered positions are shown.

Due to the computational expense involved and the prevailing view that they are not significantly involved in the electronic transitions, alkyl chains are often excluded when modelling the electronic properties of Y6 crystals or films. The fact we are able to model them validates the higher quality of these metallic red crystals.[6] Furthermore, it allows insight into how the alkyl chains affect packing.

The molecules in adjacent columns are 6.7 Å apart, which is too far for significant orbital overlap. The molecules in the same column overlap significantly— i.e., the undecyl alkyl

chains on each molecule are positioned for van der Waals interactions with the branched alkyl chains on the molecule in front, effectively filling the space available (**Figure S11**). This is likely to provide some additional stabilization, and by reducing the disorder in the alkyl chains may help explain why the crystals are able to grow more easily. Where the distance between the alkyl chains is greater than the van der Waals distance, the primary reason for their interaction may be a sort of 'hydrophobic effect'— avoiding disruption of backbone-solvent and solvent-solvent interactions.

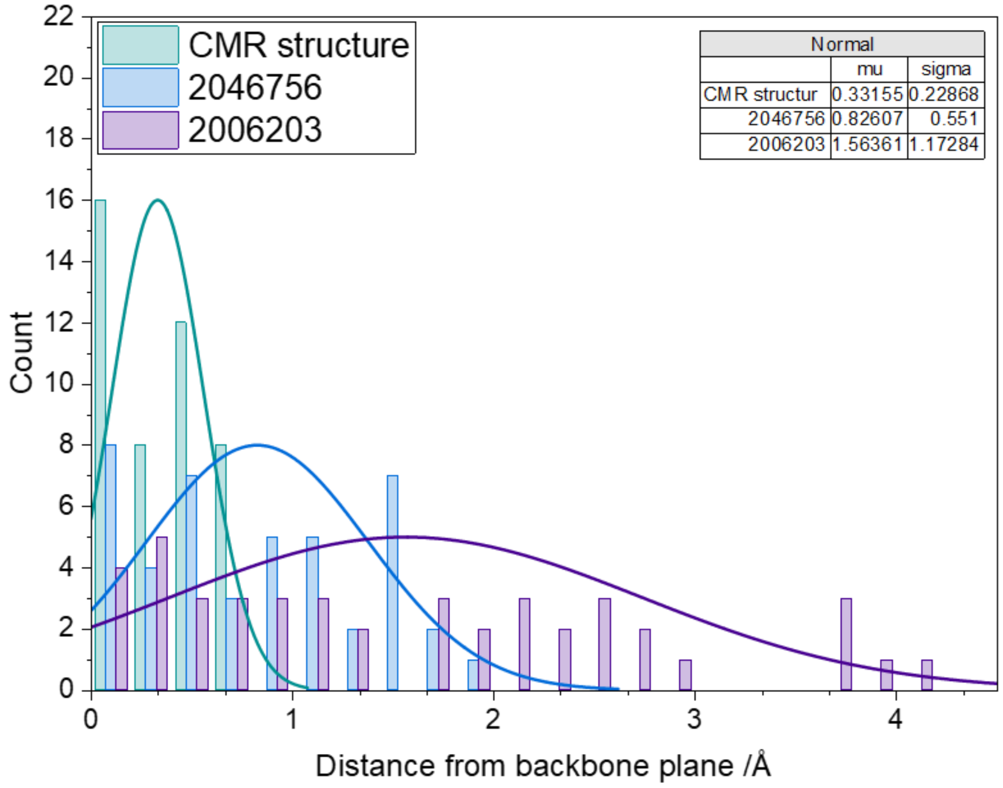

Figure S12. A binned histogram showing the distribution of distances from the atoms in the undecyl chain to the backbone plane. Disordered positions are averaged. CMR is the name used to distinguish the metallic red crystals from the literature values

To compare the modelling of the alkyl chains, 2015912 cannot be used as the structure lacks most of the relevant atoms;[7] thus, it is replaced here by 2006203 (W. Zhu et al).[8] In MRC

structure, the linear alkyl chains are very much in the plane of the backbone (the average distance to the backbone plane is 0.33 Å, and all are less than 1 Å). In contrast, 2006203 and 2046756 have modelled alkyl chains much further out of the backbone plane (**Figure S12**). By analogy with the MRC structure, one may expect shortening the linear alkyl chains to lead to denser packing in the columns, while shortening the branched alkyl chains may lead to denser interplanar stacking. However, analysis based on other polymorphs could lead to the reasonable assumption that shortening the linear alkyl chains also leads to denser π-π stacking (as these are out of plane too).

	S1	N1	C1	C2	C3	N2	C4	C5	S2	C6	C7	S3	C8	C9	C10	H10	C11	C12	O1	C13	C14	H14	C15	F1	C16	F2	C17	H17	C18	C19	C20	C21	N3	C22	C22-2	N4	N4-2
S1																											4.28	3.74						4.45		4.06	
N1																											3.80	3.42	4.19	4.35	4.15			4.50	3.69	3.86	3.70
C1																											4.48	3.96		4.49	3.91	4.41		3.40	3.94	3.48	3.36
C2																														4.29	3.59	3.68	4.10	3.51	4.34	3.84	3.79
C3																															4.21	3.86	3.92	4.08	4.35	4.15	4.25
N2																																3.92	3.67				
C4															4.46	4.06	4.43													3.96	3.55	3.49	3.83	4.01			
C5																4.15															4.22	3.68	3.61				
S2															3.76	3.74	3.62	4.27											4.17	3.58	3.81	4.04					
C6															4.37	3.93									4.49	3.95						4.23	4.05				
C7													4.13	4.04	3.73	3.58	4.19									4.12						4.35	4.47				
S3													4.43										4.41		3.76	3.73	3.84	3.84									
C8											4.13	4.43	3.57	3.66	3.86	3.91							4.20	4.00	4.28	4.10											
C9											4.04		3.66	4.24							4.21		3.69	3.94	3.65	3.82	4.13										
C10							4.46		3.76	4.37	3.73		3.86							3.89	3.57	3.84	3.51	3.92	3.85	4.44	4.21		4.23								
H10							4.06	4.15	3.74	3.93	3.58		3.91							4.20	3.62	3.67	3.61	3.76	4.25												
C11							4.43		3.62		4.19						4.40	3.84	4.23	3.40	3.50	3.77	3.89		4.19		4.15		3.75	4.34							
C12									4.27								3.84	3.78	4.43	3.51	4.09		4.49		4.39		3.90	4.28	3.40	3.58	4.17						
O1																	4.23	4.43		3.95					4.22		3.58	3.70	3.41	3.56	3.79			3.85		4.33	
C13															3.89	4.20	3.40	3.51	3.95	3.86									3.92	3.64	4.25						
C14														4.21	3.57	3.62	3.50	4.09											4.48	3.77	4.02	4.10					
H14															3.84	3.67	3.77													3.71	3.65	3.62	4.00	4.38			
C15												4.41	4.20	3.69	3.51	3.61	3.89	4.49																			
F1													4.00	3.94	3.92	3.76																					
C16										4.49		3.76	4.28	3.65	3.85	4.25	4.19	4.39	4.22																		
F2										3.95	4.12	3.73	4.10	3.82	4.44																						
C17	4.28	3.80	4.48									3.84		4.13	4.21		4.15	3.90	3.58																		
H17	3.74	3.42	3.96									3.84						4.28	3.70																		
C18		4.19							4.17						4.23		3.75	3.40	3.41	3.92	4.48								4.45	4.37							
C19		4.35	4.49	4.29			3.96		3.58								4.34	3.58	3.56	3.64	3.77	3.71							4.37								
C20		4.15	3.91	3.59	4.21		3.55	4.22	3.81									4.17	3.79	4.25	4.02	3.65															
C21			4.41	3.68	3.86	3.92	3.49	3.68	4.04	4.23	4.35										4.10	3.62															
N3				4.10	3.92	3.67	3.83	3.61		4.05	4.47											4.00															
C22	4.45	4.50	3.40	3.51	4.08		4.01												3.85			4.38															
C22:2		3.69	3.94	4.34	4.35																																
N4	4.06	3.86	3.48	3.84	4.15														4.33																		
N4:2		3.70	3.36	3.79	4.25																																

Figure S13. Interatomic distances in the MRC structure ≤ 4.5 Å

Due to the high symmetry of the structure, there is only 1 value for each distance. The colour of the text used for the atom label indicates the part of the molecule; blue = A, purple = A'. Pair 2 is responsible for the clusters with the core-core interactions and the closest core-terminal distances, while Pair 1 is responsible for the remaining distances.

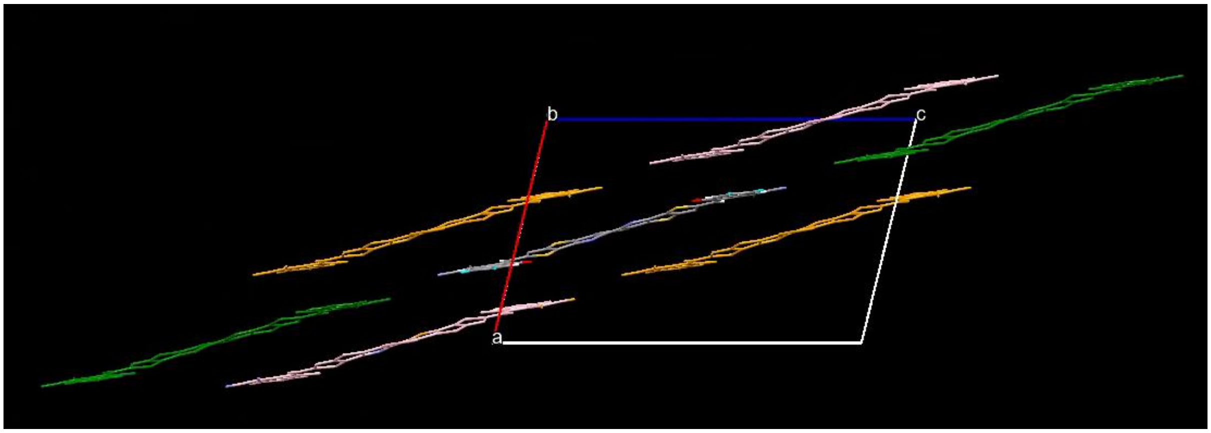

Figure S14. A view down the -b axis of the crystal (alkyl chains omitted for clarity). All molecules are symmetry-equivalent, but to aid the eye colours have been used; green molecules are those in the same plane as the central molecule, yellow molecules are those forming Pair 1 (core-core or core-terminal) interactions with the central molecule, and pink molecules are those forming Pair 2 (mainly terminal-terminal) interactions.

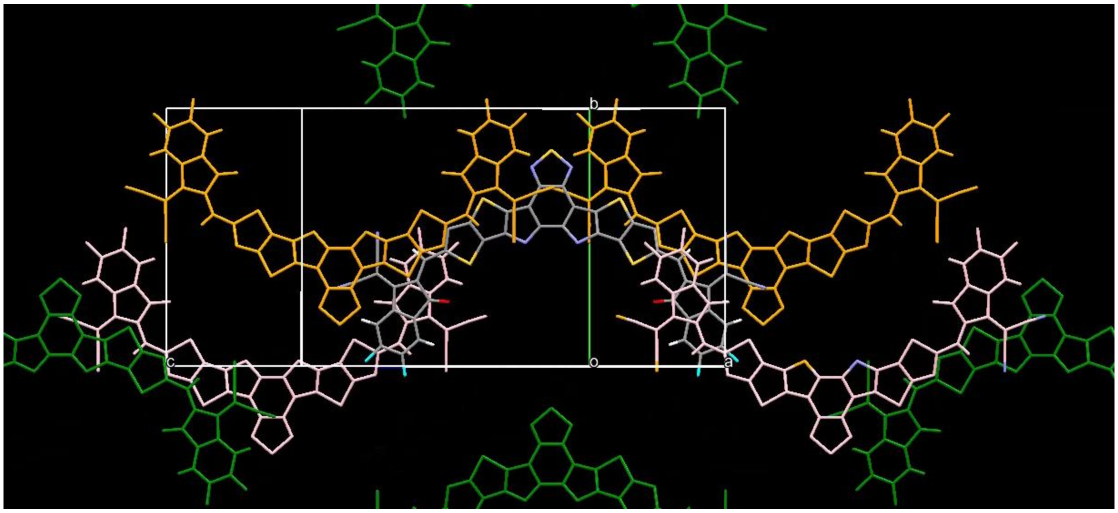

Figure S15. A view perpendicular to the molecular backbone plane

Section 4: DFT calculations

To understand the photophysical properties of the MRC structure, density functional theory calculations of Frenkel exciton – charge-transfer (FE-CT) couplings were carried out.9 The high symmetry of the structure means there are only two unique dimers with π-π stacking interactions (Figure S14). Previous authors have used an interatomic distance of 5 Å,10 or the sum of the van der Waals radii + 0.4 Å,11 as cut-offs to choose whether to evaluate the coupling strength of dimers. In this structure, only the pairs mentioned have backbone distances in that range.

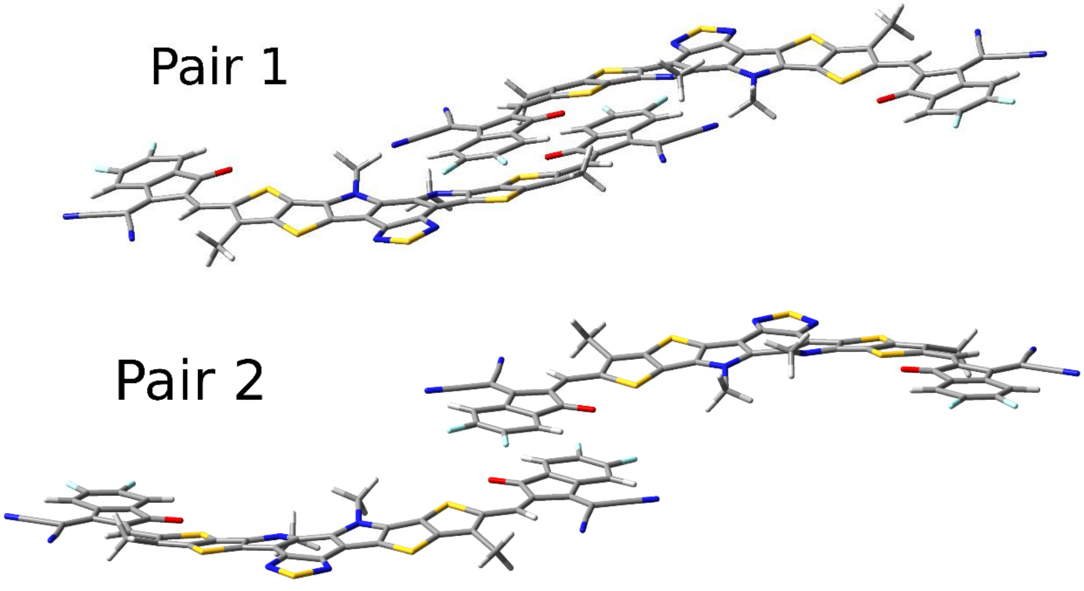

Figure S16. Dimer pairs selected for DFT calculations. Alkyl chains were truncated to methyl to reduce computational cost. Methodology reported in section 4 of the main text.

Table 2: Energy levels and coupling magnitudes for excited states in dimers extracted from the MRC structure. The values in grey are equivalent to those elsewhere in the table.

Dimer 1

	E/ eV	$\|V\|$ /meV			
		FE_1	FE_2	CT_1	CT_2
FE_1	1.64	-	4	41.9	14.4
FE_2	1.64	4	-	14.4	41.9
CT_1	1.56	41.9	14.4	-	1
CT_2	1.56	14.4	41.9	1	-

Dimer 2

	E/ eV	$\|V\|$ /meV			
		FE_1	FE_2	CT_1	CT_2
FE_1	1.64	-	33	31	50
FE_2	1.64	33	-	50	31
CT_1	1.61	31	50	-	3
CT_2	1.61	50	31	3	-

The maximum FE-CT couplings are 42 meV in Pair 1 and 50 meV in Pair 2, and there is close energetic alignment between the localised FE state and the intermolecular CT state. FE1 and FE2 differ in which molecule of the dimer the Frenkel exciton is located on, while CT1 and CT2 differ in which molecule bears the hole (or the electron). Due to the high symmetry of the crystal the energy levels of these states are equal.

There is good agreement between the DFT calculations carried out here and the DFT calculations previously carried out on 2015912.[7] The FE state is slightly lower in energy than the states present in the 2015912 dimers, which have energies of 1.71-2.04 eV, but are still higher in energy than the CT states, as was true for most dimers in the previous work. The calculated FE CT couplings in

the work by Price et al. include values of 37, 36 and 75 meV (for the FE to CT states where the Frenkel excitons are higher in energy than CT states).[12] Thus the conversion of Frenkel excitons to a charge transfer state (the precursor to free charge generation) appears both probable and energetically favourable in both structures.

Section 5: Micro-ellipsometry

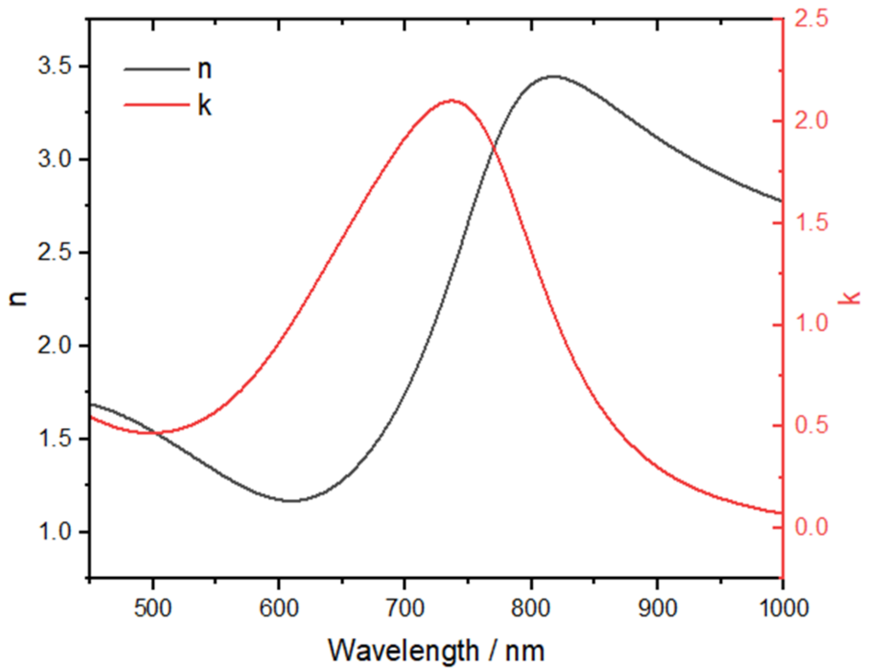

Figure S17. Micro-ellipsometry of metallic red Y6 crystals, showing the wavelength dependence of the real (n) and imaginary (k) components of the refractive index.

Experimental details for the micro-ellipsometry measurements are provided in the Methods section of the main text. At 800 nm, the magnitude of k shows agreement with previously reported literature values for Y6 thin films,[2] though the spectral shape is shifted slightly. Spectral deviations are expected as a result of the difficulty in measuring micro-millimetre-sized samples, and differences in instrumentation.

Section 6: Calculation of photo-excited carrier densities

The density of optical excitations within thick crystals is often modelled by assuming a thickness equal to the optical penetration depth. However, in our case this assumption will lead to an overestimation of the carrier density (hence underestimation of photocarrier diffusion length), as it does not account for the 37% of light that penetrates beyond this distance. Therefore, a fuller treatment of absorption and reflection (similar to Burkhard et al.) is required.[13]

Based on our micro-ellipsometry (**Supplementary Information Section 5**) measurement, absorption and reflection coefficients are similar in magnitude to those reported by Kerremans et al.[2] This agreement is also confirmed by rough measurements of the reflected power from the crystals and films at 800 nm, which show a 10-14% reflection at an incidence of 65 degrees, consistent with Fresnel expressions for P-polarised light using optical constants similar to those of Kerremans et al.[2]

After reflection, the absorption of the Y6 samples is modelled by a formula modified from the standard expression for estimated density in a thin film, where we conceptually split the crystal into multiple thin 'slices', and sum the photoluminescence contribution from each slice (with its own estimated carrier density) to obtain the full equivalent measured signal.

Considering the Beer-Lambert law, the photoexcited carrier density for each of the *n* slices of thickness Δx is:

$$\rho_n = \frac{I_0 e^{-\alpha n \Delta x}(1 - e^{-\alpha \Delta x})}{\Delta x} \tag{1}$$

Where I_0 is the number of photons incident on the sample per square meter, α is the absorption coefficient of the sample, Δx is the thickness of the slice, and n is the index of the 'slice', from $n = 0$ to $n = n_{max}$, where n_{max} is equal to the total sample thickness divided by Δx.

The measured PL efficiency, η, as a function of incident photon density, I_0, is then:

$$\eta(I_0) = \sum_{n=0}^{n_{\max}} \frac{1}{\rho_n} e^{-\alpha n \Delta x} \int_{t=0}^{t_\infty} k_{\mathrm{rad}} \mathrm{S}(\rho_n, t) \tag{2}$$

Where $\mathrm{S}(\rho_n, t)$ is the singlet exciton population as a function of time, t, and excitation density, for each slice, calculated according to the systems of ordinary differential equations detailed in **Supplementary Information Section 7**, and multiplied by the radiative excitonic decay rate coefficient, k_{rad}.

We note that if the measured material shows a significant PLQY (above ~5%), then care must also be taken to account for photon reabsorption and subsequent photon recycling in addition to the Beer-Lambert model considered above, as this photon recycling can lead to increased carrier densities which would result in higher apparent diffusion coefficients, if not accounted for.[10]

Section 7: Kinetic models of pulsed PLQY

We present here the three example kinetic models used to analyse the PPLQY data for thin films and crystals.

Model 1

This model uses the standard expression for rate of change of the singlet state population,

$$\frac{dS}{dt} = -kS - \gamma S^2 \tag{3}$$

Where k and γ are the rate coefficients for unimolecular radiative recombination and bimolecular nonradiative annihilation, and $\frac{dS}{dt}$ represents the change of singlet population with respect to time. S is used to denote the singlet state, but it should be noted that while this state is of singlet character, there is good evidence that it is a delocalized, hybrid Frenkel-CT state upon photoexcitation.

Fitting the data to this model is done by minimising the least squares error (see **Figure S17**).

Model 2

$$\frac{dS}{dt} = -kS - \gamma S^2 + \frac{1}{2}k_{\mathrm{tta}}T^2, \tag{4}$$

$$\frac{dT}{dt} = k_{\mathrm{isc}}S + k_{\mathrm{st}}S^2 - k_{\mathrm{T}}T - k_{\mathrm{tta}}T^2, \tag{5}$$

Here, $\frac{dS}{dt}$ and $\frac{dT}{dt}$ represent the changes in singlet and triplet state populations with respect to time, k is the unimolecular radiative recombination rate coefficient, γ is the bimolecular nonradiative annihilation rate coefficient, and k_{tta}, k_{isc}, and k_T are the triplet-triplet annihilation, intersystem crossing, and triplet nonradiative decay rate coefficients. k_{st} describes the rate of triplet generation achieved from the process of singlet-singlet annihilation. The triplet state population could be generated directly through this process, or likely through an intermediary such as free-charges (generated by singlet-singlet annihilation) which recombine in the expected 3:1 ratio of triplet to singlet states. Without this bimolecular triplet-state generation term, the rate of intersystem crossing required to generate enough triplet state density to account for the high fluence deviation is unphysical.

The parameters in Models 2 and 3 are constrained such that the fitted diffusion length from Model 1 is consistent across the models. This can be seen in the good fit and agreement of each model at lower fluences (**Figure 4d** main text). While these parameters are physically reasonable, the larger number of parameters in these models means that the value of these parameters presented below do not represent fitted values.

Table 3: Rate coefficients used in the main text for models 2 and 3.

Definition	Symbol	Derived	Value
Radiative decay rate coefficient	k_{rad}		2.3×10^{8} s^{-1}
Intersystem crossing rate coefficient	k_{ISC}		1.0×10^{8} s^{-1}
Non-radiative decay rate coefficient	k_{NR}		6.7×10^{8} s^{-1}
Singlet-state unimolecular loss rate coefficient	k_{UL}	$k_{rad} + k_{ISC} + k_{NR}$	1.0×10^{9} s^{-1}
Singlet-singlet annihilation rate coefficient	$\mathrm{k_{SSA}}$		1.5×10^{-8} s^{-1}
Triplet-triplet annihilation rate coefficient	k_{TTA}		0.8×10^{-11} s^{-1}
Triplet state non-radiative decay rate coefficient	k_{T}		1.0×10^{4} s^{-1}
Triplet state generation through singlet-singlet annihilation	k_{TS}		3.8×10^{-11} s^{-1}
Singlet state lifetime	τ	$\frac{1}{k_{UL}} \times 10^{9}$	~1.0 ns
Exciton annihilation radius	R_0		1.5 nm
Diffusion length	L_D	$\sqrt{\frac{6*k_{SSA}*\frac{1}{k_{UL}}*1\times 10^{-6}}{4\pi*1.5\times 10^{-9}}} \times 10^{9}$	~70 nm

The increased parameters for Model 2 mean the parameters are interdependent, so errors on individual parameters are not included.

The annihilation radius here for all models is assumed to be 1.5 nm, for easy comparison to literature values.[14] This value is derived from the 'd_{100}' spacing of GIWAXS measurements on neat films and implicitly assumes that the excitons are delocalized across the unit cell. We stress that this value is used purely for comparison to thin films of Y6 measured by the same method.[14] It is also common to use 1 nm as an annihilation radius. A physically more meaningful radius would be an estimated self-Förster radius, as this likely represents the hopping distance of singlet excitons. However, this choice will have a large error associated with the delocalized nature of Y6 excitons and the difficulty in estimating the true absorption strength of the Y6 crystals.

Model 3

This is the model used by Price, Hume, *et al.*[10] The full details of this model can be found in reference [6]. Due to the larger number of free parameters, we do not consider this model to be a quantitative fit to the current data. It is used merely to illustrate that if the singlet diffusion length is constrained to the value found by fitting this and the other models to the low fluence portion of the data (as shown in main text **Figure 4 d**), a good fit to the higher fluence data can also be obtained.

The kinetic equations are given below:

$$\frac{dS^*}{dt} = -(k_{\text{relax}} + k_{\text{cs}})S^* - \gamma S^*(S^* + S) + \frac{1}{2}k_{\text{tta}}T^2 \tag{6}$$

$$\frac{dS}{dt} = k_{\text{relax}}S^* + \frac{1}{4}k_{\text{enc}}C_e C_h - kS - \gamma S(S + S^*) \tag{7}$$

$$\frac{dC_e/C_h}{dt} = k_{\text{cs}}S^* - k_{\text{enc}}C_e C_h - k_{\text{srh}}(C_e/C_h)Q \tag{8}$$

$$\frac{dT}{dt} = \frac{3}{4}k_{\text{enc}}C_e C_h - k_{\text{tta}}T^2 - k_{tc}T(C_e + C_h) \tag{9}$$

$$\frac{dQ}{dt} = -k_{\text{srh}}(C_e/C_h)Q \tag{10}$$

Where, S^* is a population of photoexcited delocalized exciton states. S are singlet excitons, C_e/C_h are the population of electrons/holes respectively (constrained here to be of equal concentrations), T is the triplet-state population, and Q is the trap population (to simplify, we set this to zero, so the trap population is implicitly assumed to be static). γ is the S and S^* bimolecular annihilation rate coefficient, k_{relax} is the rate coefficient for relaxation from S^* to S, k_{cs} is the rate coefficient for separation from S^* to charges, k_{enc} is the rate coefficient for electron-hole encounters, k_{srh} is the Shockley-Read-Hall recombination rate coefficient, k_{tta} is the triplet-triplet annihilation rate coefficient, k_{tc} is the triplet-charge annihilation rate coefficient.

Section 8: Error analysis and kinetic model bounds

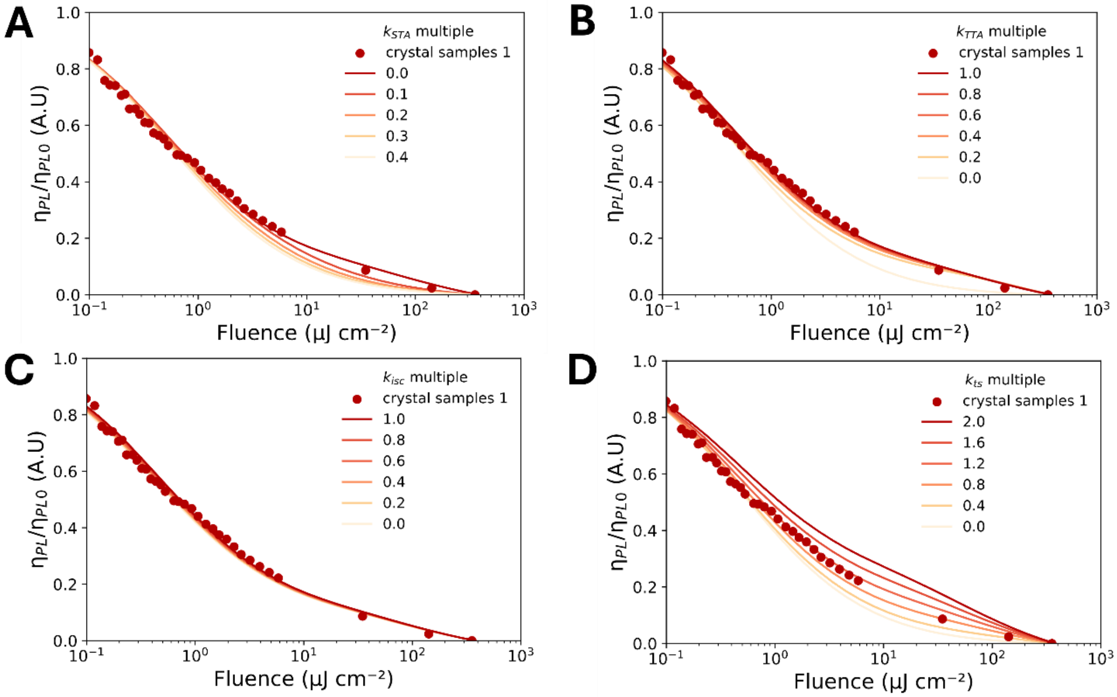

Figure S18. The effect of adjusting A) the rate coefficient of singlet-triplet annihilation, B) the rate coefficient of triplet-triplet annihilation, C) the rate coefficient of intersystem crossing, D) the rate coefficient of triplet generation through singlet-singlet annihilation. The insets in each of the figures represent the value the rate coefficients were multiplied by, to show the effect of varying the coefficient from the fitted value.

Figure S18 (A) shows the effect on Model 2 with the inclusion of singlet-triplet annihilation as a pathway for singlet and triplet decay, with the inset representing the value the rate coefficient was multiplied by. The plot of 0.0 represents Model 2 with no singlet-triplet annihilation pathway. **Figure S18 (B-D)** shows the effect of varying the different parameters in Model 2 over a wide range of values, with the inset representing the value the rate coefficient was multiplied by. The figure illustrates that varying these parameters has little effect on the low fluence fit of the model to the data.

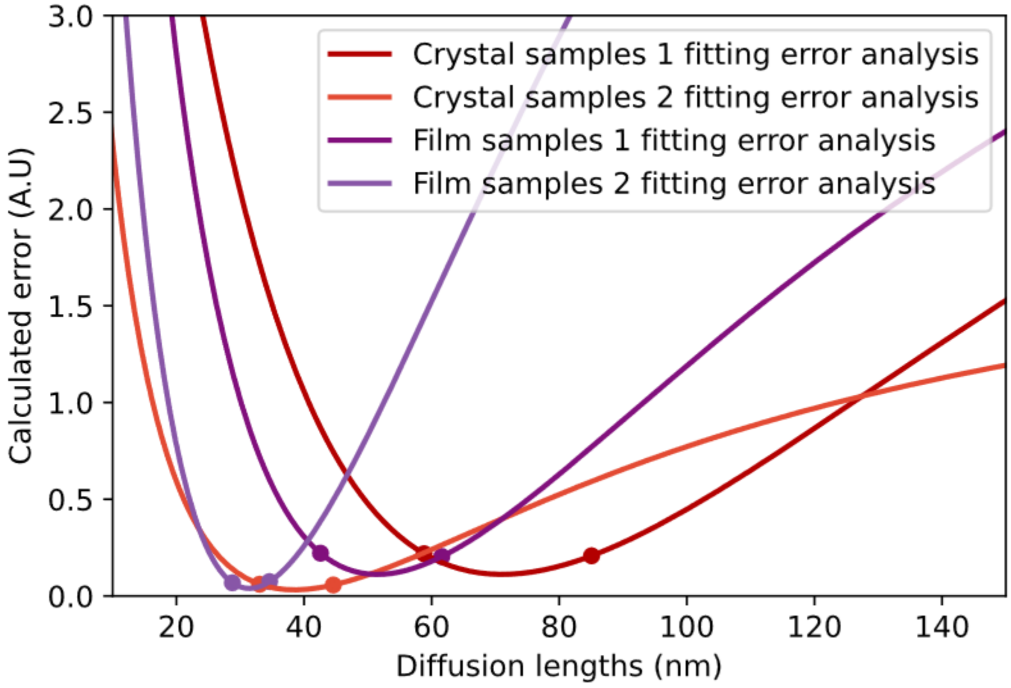

Figure S19. The least-squares error achieved when fitting model 1 to pulsed-PLQY data. Small circles are shown on each line to represent the upper and lower errors calculated for each fit.

Error values quoted in the main text are derived from double the minimum least squares error above and below the calculated model value. The calculated upper and lower errors are shown in **Figure S19** as small circles on the line. The error spread can be reduced further when only considering the low fluence region where the triplet-state contributions are minimal, but the full fluence region fit errors are shown here.

Section 9: Thermal and photostability measurements

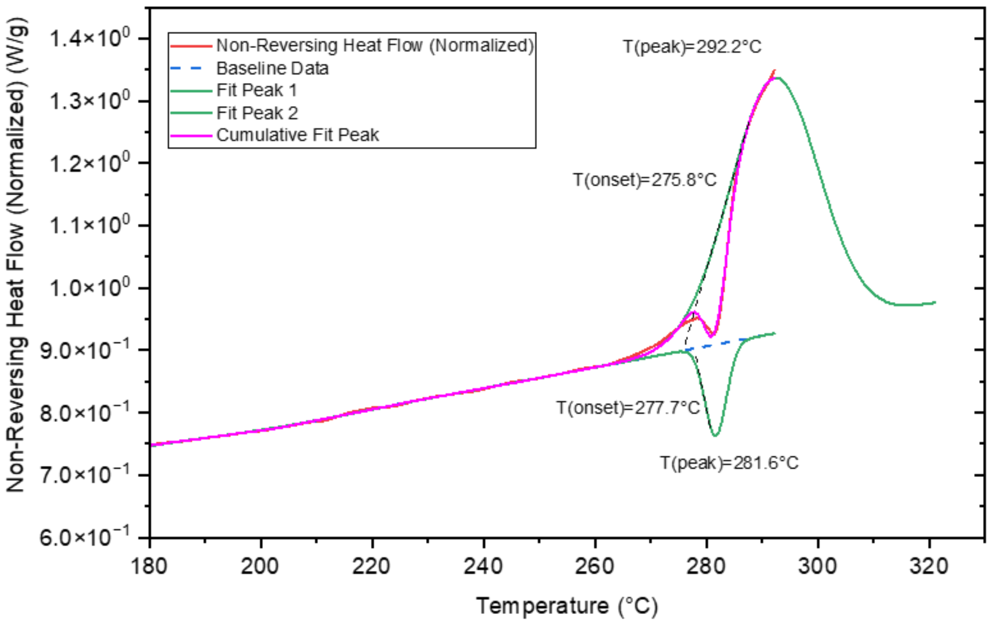

Figure S20. M-DSC thermogram of Y6 crystals grown by convection

Differential scanning calorimetry (DSC) data for crystals grown by convection (**Figure S4**) showed the absence of phase transitions below 277.7 °C— at this temperature an exothermic process appears to begin (most likely decomposition), followed by an endothermic process at 281.6 °C. The overlap between these peaks makes them harder to characterize, but the presence of the melting peak in both the non-reversing and reversing heat flow allows the peaks to be decomposed with more confidence. As the decomposition is irreversible and thus requires the sacrifice of crystals, limited trials were run.

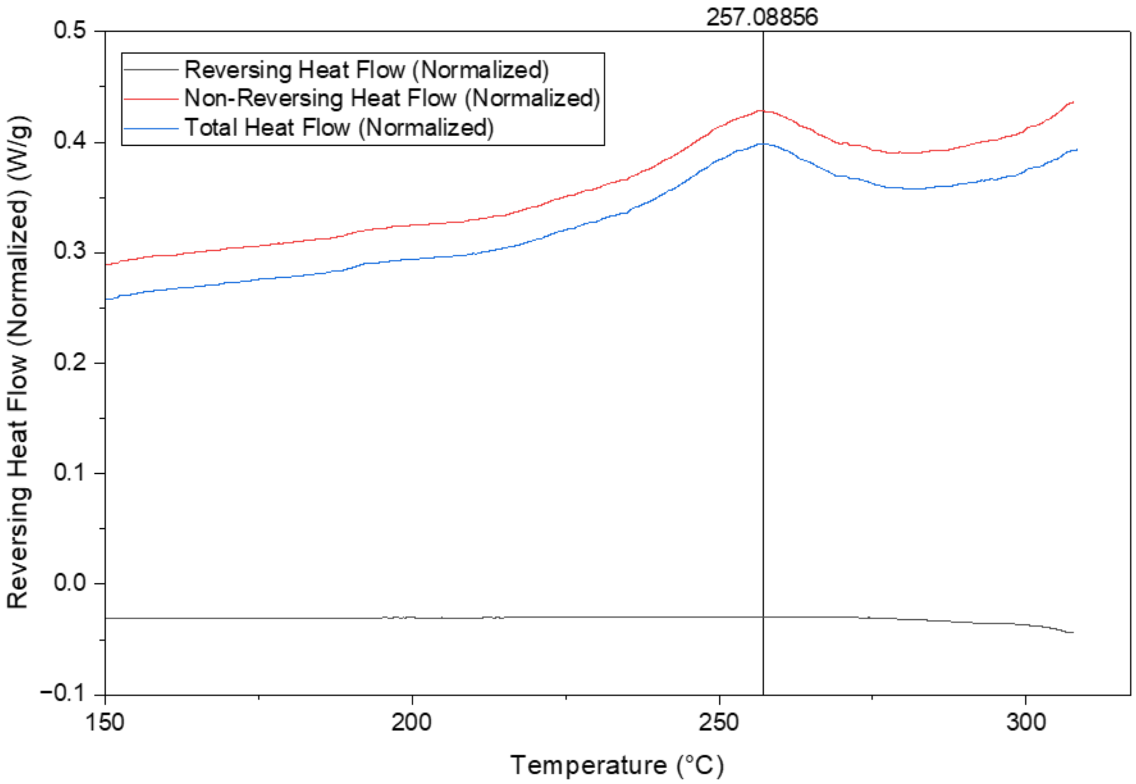

Figure S21. A cold crystallization at 257 ºC. The absence of any peaks in reversing heat flow confirms the absence of a melting process before the onset of decomposition

Under similar conditions, the powder seemed to show a cold crystallization (crystallization that occurs on heating, from a non-equilibrium state) at ~257 °C, and no melting peak before the onset of decomposition (the absence of a peak in the reversing heat flow confirms the peak is not, for example, another decomposition process). At a higher ramp rate, the amorphous powder showed a weak exothermic transition at ~235 °C, immediately followed by an endothermic peak. This is hard to interpret; it could possibly be a glass transition followed by enthalpic recovery, although this is not clear from the data. More importantly, despite the absence of an exothermic crystallization peak, there seems to be a melting peak at 295 °C, which overlaps with a higher

temperature exothermic process— this higher melting temperature could imply that the crystals grown by convection are less stable than crystals grown from the melt.

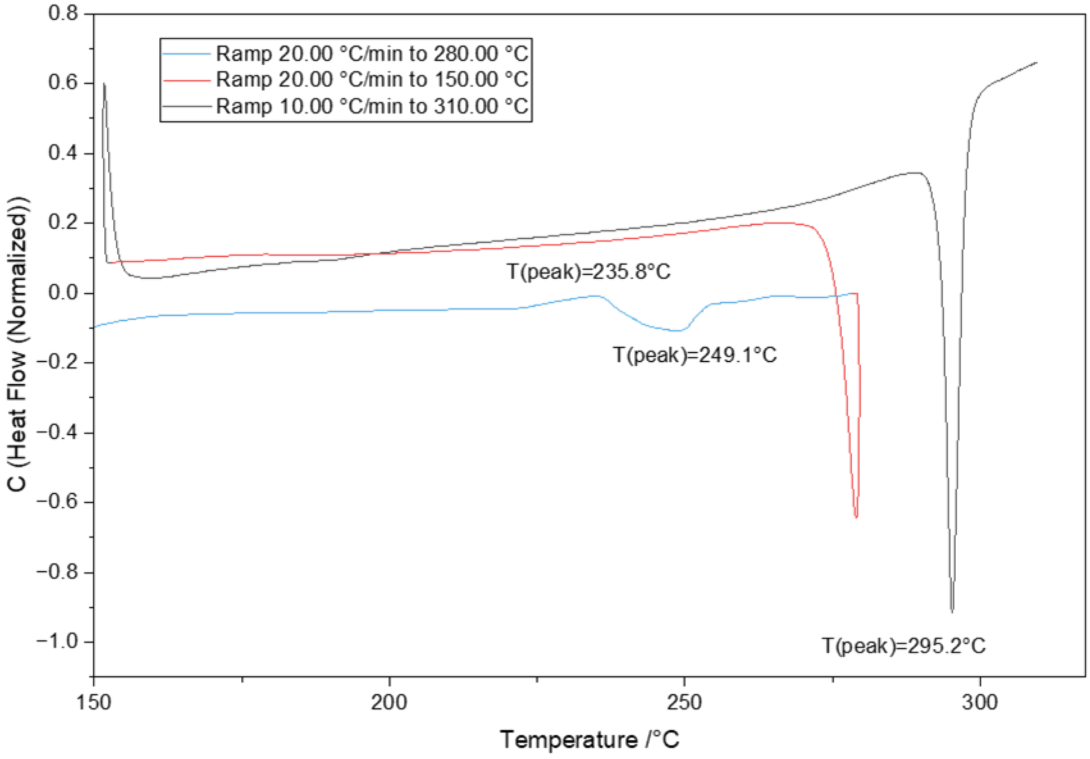

Figure S22. DSC thermogram of Y6 powder, showing a strong endothermic peak at ~295 ºC. The increased gradient of the heat flow reflects the beginning of the exothermic decomposition, but the exact onset point is difficult to discern

The DSC thermogram in **Figure S22** highlights the difficulty in carrying out DSC on a material that exhibits a weak tendency to crystallize, diverse polymorphism, and a decomposition range overlapping with its T_m. The measurement of a higher melting temperature could be indicative of an as-yet undiscovered polymorph that is more stable than the crystals grown by convection.

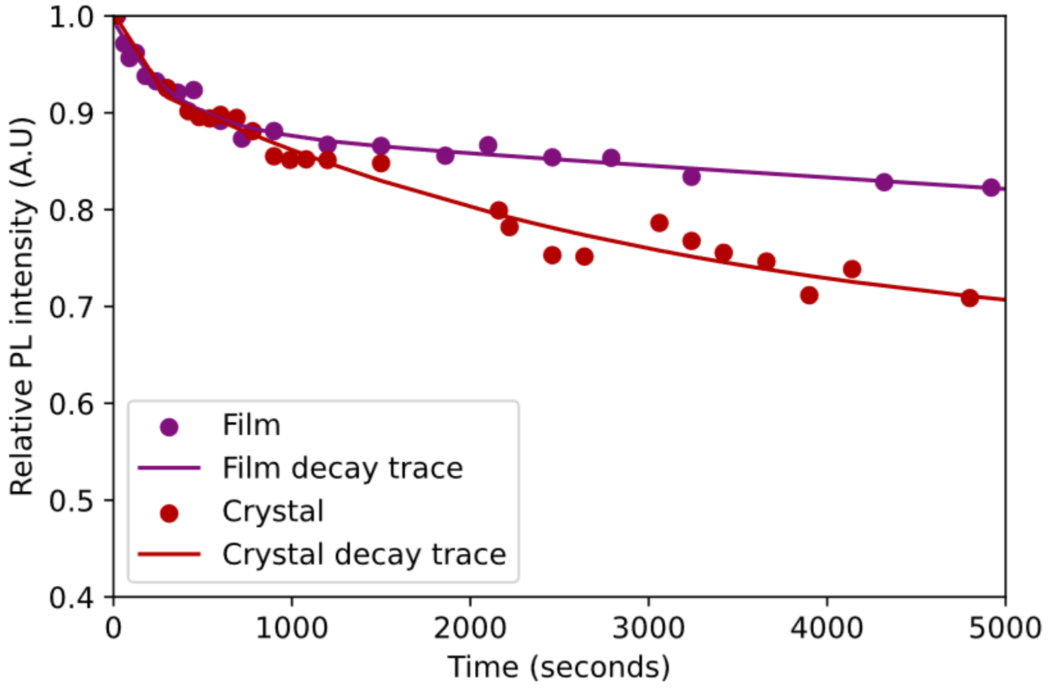

Figure S23. Photostability measurement taken over the course of several hours. Scatter plots represent individual datapoints, line plots represent the observed trends.

Samples were unenclosed and exposed to a high-fluence 800 nm wavelength beam, with the PL spectra being recorded periodically, to show the degradation of the materials with respect to time in oxygenated environments. Both film and crystal samples showed an immediate drop in a matter of seconds in overall efficiency, followed by a slow drop over the course of several hours. After an hour of continuous exposure, the film sample PL efficiency had dropped by roughly 15%, and the crystal sample PL efficiency by roughly 25%.

Table 4: Lifetimes and weights extracted from biexponential fitted FLIM data.

	Weighted τ (ns)	τ_1 (ns)	β_1	τ_2 (ns)	β_2
Crystal samples 1	1.24 (± 0.04)	0.26 (± 0.01)	0.26 (± 0.02)	1.59 (± 0.03)	0.73 (± 0.01)
Crystal samples 2	0.83 (± 0.04)	0.18 (± 0.01)	0.30 (± 0.02)	1.14 (± 0.04)	0.64 (± 0.02)
Film samples	1.07 (± 0.04)	0.28 (± 0.02)	0.22 (± 0.02)	1.3 (± 0.02)	0.79 (± 0.02)

τ_1, and τ_2 are the extracted lifetimes. β_1, and β_2 are the weights of each extracted lifetime.

Table 5: Lifetimes and weights extracted from triexponential fitted FLIM data.

	Weighted τ (ns)	τ_1 (ns)	β_1	τ_2 (ns)	β_2	τ_3 (ns)	β_3	χ^2
Crystal samples 1	1.26	0.07	0.93	0.95	0.06	3.49	0.01	6.09
Crystal samples 2	0.44	0.05	0.99	0.78	0.01	6.51	0.00	12.68
Film samples	1.24	0.05	0.91	1.01	0.09	4.14	0.01	35.75

τ1, τ2, and τ3 are the extracted lifetimes. β1, β2, and β3 are the weights of each extracted lifetime. χ^2 is the chi-squared value for the fits obtained from the FLIMfit software.

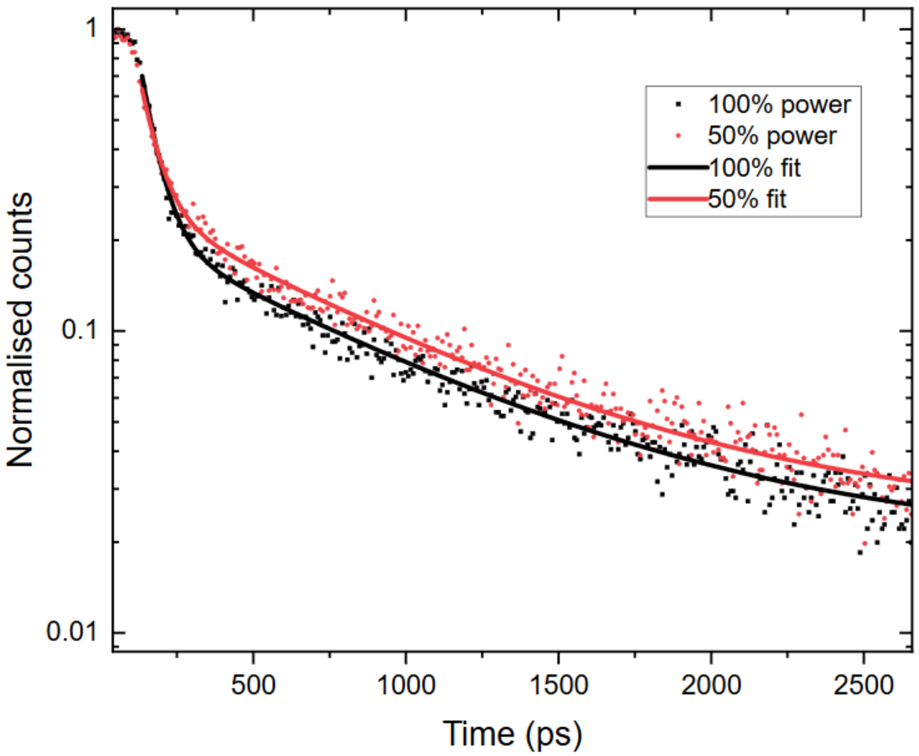

Figure S24. Weak intensity dependence of the fast component of crystal samples photo-excited state lifetimes as observed in FLIM

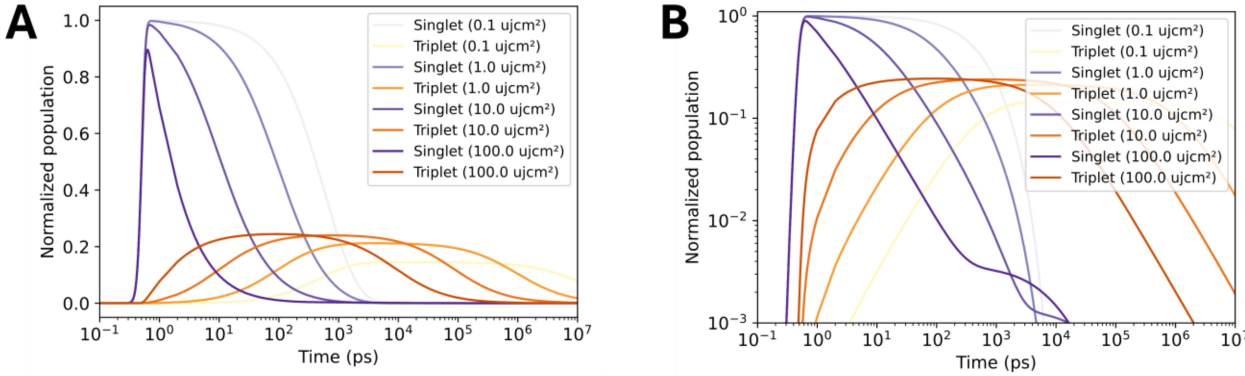

Figure S25. A) Linearly and B) logarithmically scaled modelled normalized excited-state populations. The maximum triplet-state population increases from 10-25% moving from simulated low to high fluence, while the singlet-state population decay kinetics show slight

changes at 1 ns coming from triplet-triplet annihilation repopulating the singlet state (seen most clearly by the dark blue singlet-state decay trace on a log scale).

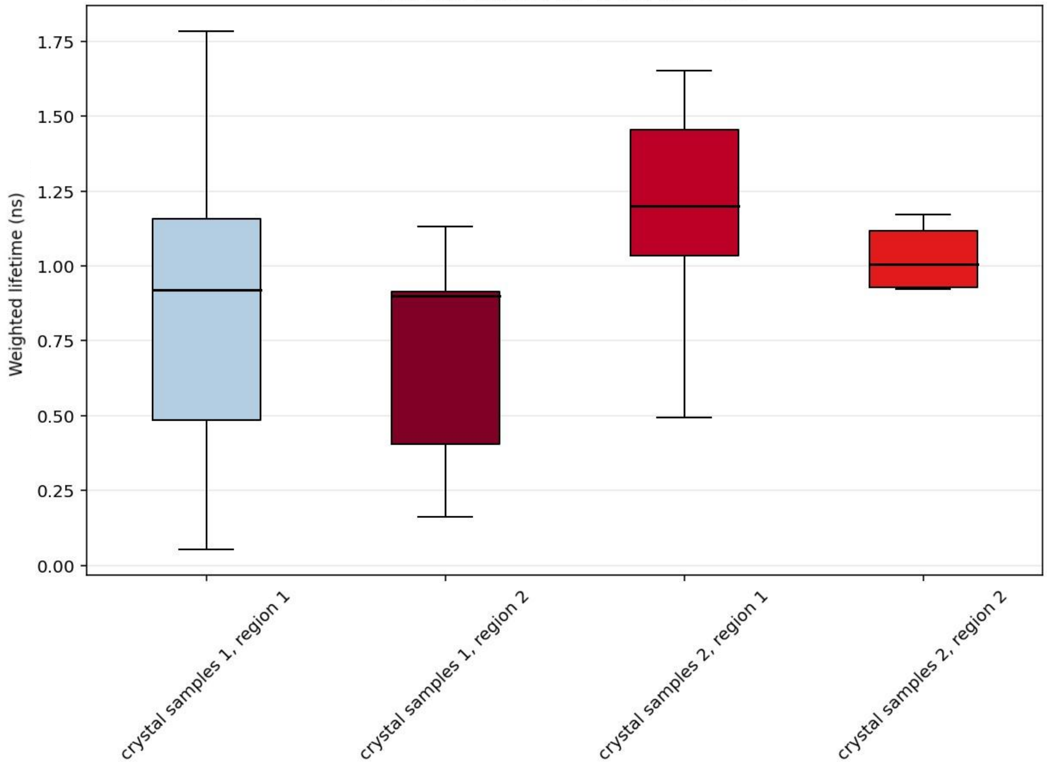

Figure S26. Intrasample variance of weighted lifetimes for different MRC batches and regions, obtained from fluorescence lifetime imaging microscopy (FLIM). Data were normalized at 500 ps and fit to a biexponential model. This analysis illustrates the degree of variation across crystallization batches, and across different crystalline regions. The distribution is skewed towards un-physically short lifetimes because different crystal heights give rise to greater pump scatter compared to the fluorescence contributions.

Section 10: Solvent volatility analysis

Table 6: Chemicals used as processing solvents or additives for Y6, and their vapour pressures at room temperature.

Chemical	Vapour pressure at 25 °C/ mmHg	Reference
Chloroform	200	[15]
Dibromomethane	40	[16]
Chlorobenzene	12	[17]
Anisole	3.54	[18]
Benzaldehyde	1.27	[19]
Acetophenone	0.372	[20]
Phenol	0.35	[21]
Naphthalene	0.085	[22]
1,4-dibromobenzene	0.058	[23]
1-chloronapthalene	0.029	[24]
Trimethoxybenzene	0.024	[25]
2-methoxynapthalene	0.00823	[26]
Ferrocene	0.00731	[27]
Tribromobenzene	0.00150012	[28]
1,8-diiodooctane	3.61×10^{-5}	[29]
Anthracene	2.67×10^{-6}	[30]
Dibenzoylmethane	1.63×10^{-6}	[31]
Oxybenzone (2-HM)	1.42×10^{-6}	[32]

AUTHOR INFORMATION

Corresponding Authors

E-mail: michael.price@bristol.ac.uk, simon.hall@bristol.ac.uk

Author Contributions

The manuscript was written through contributions of all authors. All authors have given approval to the final version of the manuscript. ‡These authors contributed equally.

ABBREVIATIONS

ITO, indium tin oxide; MCR, metallic red crystal; DFT, density functional theory; FE, Frenkel exciton; CT, charge-transfer; PPLQY, pulsed photoluminescence quantum yield; GIWAXS, grazing incident wide angle x-ray spectroscopy; DSC, differential scanning calorimetry; FLIM, fluorescence lifetime imaging microscopy.

REFERENCES

(1) Klebe, G.; Graser, F.; Hadicke, E.; Berndt, J. Crystallochromy as a solid-state effect: correlation of molecular conformation, crystal packing and colour in perylene-3,4:9,10-bis(dicarboximide) pigments. *Acta Crystallographica Section B* **1989**, *45* (1), 69-77.

(2) Kerremans, R.; Kaiser, C.; Li, W.; Zarrabi, N.; Meredith, P.; Armin, A. The Optical Constants of Solution-Processed Semiconductors—New Challenges with Perovskites and Non-Fullerene Acceptors. *Advanced Optical Materials* **2020**, *8* (16), 2000319.

(3) Venkatesh, V. Twinning in Cordierite. *Am Mineral* **1954**, *39* (7-8), 636-646.

(4) Chen, S. A.; Xi, H. M.; Yu, L. Cross-nucleation between ROY polymorphs. *J Am Chem Soc* **2005**, *127* (49), 17439-17444.

(5) Bond, A. D.; Boese, R.; Desiraju, G. R. On the Polymorphism of Aspirin: Crystalline Aspirin as Intergrowths of Two "Polymorphic" Domains. *Angewandte Chemie International Edition* **2007**, *46* (4), 618-622.

(6) Zhou, R.; Li, C.; Wen, Z.; Zhang, C.; Shi, Y.; Hou, H.; Chen, X.; Kang, Q.; Zhang, Y.; Yan, H.; et al. Intrinsic role of alkyl side chains in disorder, aggregates, and carrier mobility of nonfullerene acceptors for organic solar cells: A multiscale theoretical study. *Aggregate* **2025**, *6* (2), e664.

(7) Zhang, G. C.; Chen, X. K.; Xiao, J. Y.; Chow, P. C. Y.; Ren, M. R.; Kupgan, G.; Jiao, X. C.; Chan, C. C. S.; Du, X. Y.; Xia, R. X.; et al. Delocalization of exciton and electron wavefunction in non-fullerene acceptor molecules enables efficient organic solar cells. *Nat Commun* **2020**, *11* (1).

(8) Zhu, W.; Spencer, A. P.; Mukherjee, S.; Alzola, J. M.; Sangwan, V. K.; Amsterdam, S. H.; Swick, S. M.; Jones, L. O.; Heiber, M. C.; Herzing, A. A.; et al. Crystallography, Morphology, Electronic Structure, and Transport in Non-Fullerene/Non-Indacenodithienothiophene Polymer:Y6 Solar Cells. *J Am Chem Soc* **2020**, *142* (34), 14532-14547.

(9) Hume, P. A.; Hodgkiss, J. M. A Projective Method for the Calculation of Excited-State Electronic Coupling: Isolating Charge Transfer/Recombination Processes in Organic Photovoltaics. *J Phys Chem A* **2020**, *124* (3), 591-600.

(10) Price, M. B.; Hume, P. A.; Ilina, A.; Wagner, I.; Tamming, R. R.; Thorn, K. E.; Jiao, W.; Goldingay, A.; Conaghan, P. J.; Lakhwani, G.; et al. Free charge photogeneration in a single component high photovoltaic efficiency organic semiconductor. *Nature Communications* **2022**, *13* (1), 2827.

(11) Ye, L.; Weng, K.; Xu, J.; Du, X.; Chandrabose, S.; Chen, K.; Zhou, J.; Han, G.; Tan, S.; Xie, Z.; et al. Unraveling the influence of non-fullerene acceptor molecular packing on photovoltaic performance of organic solar cells. *Nat Commun* **2020**, *11* (1), 6005.

(12) Olaya-Castro, A.; Scholes, G. D. Energy transfer from Förster–Dexter theory to quantum coherent light-harvesting. *International Reviews in Physical Chemistry* **2011**, *30* (1), 49-77.

(13) Burkhard, G. F.; Hoke, E. T.; McGehee, M. D. Accounting for Interference, Scattering, and Electrode Absorption to Make Accurate Internal Quantum Efficiency Measurements in Organic and Other Thin Solar Cells. *Advanced Materials* **2010**, *22* (30), 3293-+.

(14) Riley, D. B.; Sandberg, O. J.; Li, W.; Meredith, P.; Armin, A. Quasi-Steady-State Measurement of Exciton Diffusion Lengths in Organic Semiconductors. *Phys Rev Appl* **2022**, *17* (2).

(15) National Center for Biotechnology Information. *PubChem Compound Summary for CID 6212, Chloroform. (accessed September 2025)* https://pubchem.ncbi.nlm.nih.gov/compound/Chloroform.

(16) National Center for Biotechnology Information. *PubChem Compound Summary for CID 3024, Dibromomethane. (accessed September 2025)* https://pubchem.ncbi.nlm.nih.gov/compound/Dibromomethane.

(17) Ukraintseva, E. A.; Soldatov, D. V.; Zelenina, L. N.; Plyusnin, P. E.; Ogienko, A. G. The thermodynamic stability of inclusion compounds of Zinc(II) and Nickel(II) coordination polymers with chlorobenzene as a guest: The determination of guest vapor pressure by the tensimetric method. *Russian Journal of Physical Chemistry* **2006**, *80* (12), 1920-1924.

(18) Vasiltsova, T. V.; Verevkin, S. P.; Bich, E.; Heintz, A.; Bogel-Lukasik, R.; Domanska, U. Thermodynamic Properties of Mixtures Containing Ionic Liquids. Activity Coefficients of Ethers

and Alcohols in 1-Methyl-3-Ethyl-Imidazolium Bis(Trifluoromethyl-sulfonyl) Imide Using the Transpiration Method. *Journal of Chemical & Engineering Data* **2005**, *50* (1), 142-148.

(19) Emel'yanenko, V. N.; Dabrowska, A.; Hertel, M. O.; Scheuren, H.; Sommer, K. Vapor Pressures, Enthalpies of Vaporization, and Limiting Activity Coefficients in Water at 100 °C of 2-Furanaldehyde, Benzaldehyde, Phenylethanal, and 2-Phenylethanol. *Journal of Chemical & Engineering Data* **2007**, *52* (2), 468-471.

(20) Daubert, T. E.; Danner, R. Physical and thermodynamic properties of pure chemicals : data compilation. 1989.

(21) Biddiscombe, D. P.; Martin, J. F. Vapour pressures of phenol and the cresols. *Transactions of the Faraday Society* **1958**, *54* (0), 1316-1322, 10.1039/TF9585401316.

(22) Goldfarb, J. L.; Suuberg, E. M. Vapor pressures and thermodynamics of oxygen-containing polycyclic aromatic hydrocarbons measured using knudsen effusion. *Environmental Toxicology and Chemistry* **2008**, *27* (6), 1244-1249.

(23) Solomonov, B. N.; Varfolomeev, M. A.; Nagrimanov, R. N.; Novikov, V. B.; Ziganshin, M. A.; Gerasimov, A. V.; Verevkin, S. P. Enthalpies of Vaporization and Sublimation of the Halogen-Substituted Aromatic Hydrocarbons at 298.15 K: Application of Solution Calorimetry Approach. *Journal of Chemical & Engineering Data* **2015**, *60* (3), 748-761.

(24) Lei, Y. D.; Wania, F.; Shiu, W. Y. Vapor Pressures of the Polychlorinated Naphthalenes. *Journal of Chemical & Engineering Data* **1999**, *44* (3), 577-582.

(25) The Good Scents (accessed September 2025). *1,3,5-trimethoxybenzene*. https://www.thegoodscentscompany.com/data/rw1146451.html

(26) ChemicalBook (accessed September 2025). *2-Methoxynaphthalene*. https://www.chemicalbook.com/ChemicalProductProperty_EN_CB3483471.htm

(27) Jacobs, M. H. G.; Van Ekeren, P. J.; De Kruif, C. G. The vapour pressure and enthalpy of sublimation of ferrocene. *The Journal of Chemical Thermodynamics* **1983**, *15* (7), 619-623.

(28) Huinink, J.; van Miltenburg, J. C.; Oonk, H. A. J.; Schuijff, A. Vapour-pressure measurements and thermodynamic properties; 1,3,5-tribromobenzene. *Recueil des Travaux Chimiques des Pays-Bas* **1988**, *107* (3), 273-277.

(29) Yaws, C. L.; Satyro, M. A. Chapter 1 - Vapor Pressure – Organic Compounds. In *The Yaws Handbook of Vapor Pressure (Second Edition)*, Yaws, C. L. Ed.; Gulf Professional Publishing, 2015; pp 1-314.

(30) Lei, Y. D.; Chankalal, R.; Chan, A.; Wania, F. Supercooled Liquid Vapor Pressures of the Polycyclic Aromatic Hydrocarbons. *Journal of Chemical & Engineering Data* **2002**, *47* (4), 801-806.

(31) Dykyj, J.; Svoboda, J.; Wilhoit, R. C.; Frenkel, M.; Hall, K. R. 2 Organic Compounds, C1 to C57. Part 2, pp. 111-205: Datasheet from Landolt-Börnstein - Group IV Physical Chemistry · Volume 20B: "Vapor Pressure and Antoine Constants for Oxygen Containing Organic Compounds" in SpringerMaterials

(32) National Center for Biotechnology Information. *PubChem Compound Summary for CID 4632, 2-Hydroxy-4-methoxybenzophenone. (accessed September 2025)* https://pubchem.ncbi.nlm.nih.gov/compound/2-Hydroxy-4-methoxybenzophenone.
